# The *TRIM37* variants in Mulibrey nanism patients paralyze follicular helper T cell differentiation

**DOI:** 10.1038/s41421-023-00561-z

**Published:** 2023-08-01

**Authors:** Wangpeng Gu, Jia Zhang, Qing Li, Yaguang Zhang, Xuan Lin, Bingbing Wu, Qi Yin, Jinqiao Sun, Yulan Lu, Xiaoyu Sun, Caiwei Jia, Chuanyin Li, Yu Zhang, Meng Wang, Xidi Yin, Su Wang, Jiefang Xu, Ran Wang, Songling Zhu, Shipeng Cheng, Shuangfeng Chen, Lian Liu, Lin Zhu, Chenghua Yan, Chunyan Yi, Xuezhen Li, Qiaoshi Lian, Guomei Lin, Zhiyang Ling, Liyan Ma, Min Zhou, Kuanlin Xiao, Haiming Wei, Ronggui Hu, Wenhao Zhou, Lilin Ye, Haikun Wang, Jinsong Li, Bing Sun

**Affiliations:** 1 Division of Life Sciences and Medicine, University of Science and Technology of China, Hefei, Anhui China; 2 State Key Laboratory of Cell Biology, CAS Center for Excellence in Molecular Cell Science, Shanghai Institute of Biochemistry and Cell Biology, Chinese Academy of Sciences, University of Chinese Academy of Sciences, Shanghai, China; 3 Institute of Pasteur of Shanghai, Shanghai, China; 4 School of Life Science and Technology, Shanghai Tech University, Shanghai, China; 5 Center for Molecular Medicine, Children’s Hospital of Fudan University, National Children’s Medical Center, Shanghai, China; 6 Department of Allergy and Clinical Immunology, Children’s Hospital of Fudan University, National Children’s Medical Center, Shanghai, China; 7 Department of Ophthalmology, Eye and ENT Hospital of Fudan University, Shanghai, China; 8 Department of Neonatology, Children’s Hospital of Fudan University, National Children’s Medical Center, Shanghai, China; 9 Institute of Immunology, Third Military Medical University, Chongqing, China; 10 Beijing Changping Laboratory, Beijing, China

**Keywords:** Immunology, Mechanisms of disease, Ubiquitylation

## Abstract

The Mulibrey (Muscle–liver–brain–eye) nanism caused by loss-of-function variants in *TRIM37* gene is an autosomal recessive disorder characterized by severe growth failure and constrictive pericarditis. These patients also suffer from severe respiratory infections, co-incident with an increased mortality rate. Here, we revealed that *TRIM37* variants were associated with recurrent infection. *Trim37 FIN*_*major*_ (a representative variant of Mulibrey nanism patients) and *Trim37* knockout mice were susceptible to influenza virus infection. These mice showed defects in follicular helper T (T_FH_) cell development and antibody production. The effects of Trim37 on T_FH_ cell differentiation relied on its E3 ligase activity catalyzing the K27/29-linked polyubiquitination of Bcl6 and its MATH domain-mediated interactions with Bcl6, thereby protecting Bcl6 from proteasome-mediated degradation. Collectively, these findings highlight the importance of the Trim37-Bcl6 axis in controlling the development of T_FH_ cells and the production of high-affinity antibodies, and further unveil the immunologic mechanism underlying recurrent respiratory infection in Mulibrey nanism.

## Introduction

High-affinity antibodies derived from the germinal center (GC) are critical to protective immune responses against pathogen infection^1^. The generation of high-affinity antibodies is dependent on T_FH_ cells, which are a subset of CD4^+^ T cells that supports germinal center B cell differentiation^2,3^. The transcription factor Bcl6 is the master regulator of T_FH_ cell differentiation and function. *Bcl6* deletion in CD4^+^ T cells completely abrogates T_FH_ cell differentiation and subsequent GC formation^4–6^. *Bcl6* can be upregulated by T-cell receptor (TCR) stimulation, ICOS costimulation, and CD28 costimulation, especially in a milieu with the cytokines IL-12, IL-6, and IL-21, which activate STAT1 or STAT3^2,3,7^. Many T_FH_ cell key transcription factors, including TCF1, LEF1, and BATF, positively regulate *Bcl6* transcription and T_FH_ cell differentiation^8–10^, while BLIMP1^4^, KLF2^11,12^, FOXO1^13^, and STAT5^14–16^ are negative regulators of *Bcl6* transcription and T_FH_ cell differentiation. In addition, OPN-I promotes T_FH_ differentiation by protecting Bcl6 against ubiquitin-dependent proteasome degradation^17^, indicating that post-transcriptional regulation of Bcl6 plays an important role in T_FH_ differentiation. However, the detailed mechanisms for the post-transcriptional modification of the Bcl6 protein in T_FH_ cells are not clear.

Many studies have shown that defects in T_FH_ cell differentiation or function lead to severe infections in humans. For example, mutations in *ICOSL* and *Sh2d1a*, the key genes instructing T_FH_ differentiation, result in combined immunodeficiency and mortality due to recurrent infections^18,19^. Mutations in *CD40L* and *IL21*, the key genes promoting high-affinity antibody production, manifest as severe immunodeficiency with decreased T_FH_ cells, recurrent infections, and reduced pathogen-specific antibody titers^19,20^. Hence, these studies suggest that mutations in T_FH_ cell-related key genes may be the cause of recurrent infection in patients.

To uncover the molecular mechanism of recurrent infection, we carried out whole-exome sequencing data of patients with recurrent infection and identified *TRIM37* gene variants with a statistically significant association with recurrent infection. TRIM37, an E3 ligase, contains a RING, B-box, and coiled-coil domain in the N-terminus and a specific MATH domain in the C-terminus^21^. TRIM37 is involved in many biological processes, including autophagy^22^, tumorigenesis^23,24^, post-transcriptional modification^23^, peroxisome genesis^25,26^, signal transduction^22^, and centrosome dysfunction^24,27–29^. *TRIM37* mutations in humans cause an autosomal recessive disorder named Mulibrey (Muscle–liver–brain–eye) nanism, characterized by severe growth failure and constrictive pericarditis^30^. A total of 26 mutations within the gene loci of *TRIM37* have been identified^31^, most of which occurred in patients from Finland. In addition to growth failure, a clinical review of 85 *FIN*_*major*_ (the so-called “Finnish major mutation”) patients’ hospital records from birth to diagnosis at the age of 0.02–52 years old revealed that these patients also manifest recurrent respiratory tract infections, co-incident with an increased mortality rate^32,33^. However, the mechanisms that drive recurrent respiratory tract infections remain unclear.

Here, we found that *Trim37* mutant mice were also susceptible to influenza virus infection. These mice showed T_FH_ cell differentiation and antibody production defects following vaccine immunization or influenza virus infection. We revealed that Trim37 controlled the differentiation of T_FH_ cells in a T-cell-intrinsic manner, which also relied on its E3 ligase activity and implicated a direct interaction between Bcl6 and the MATH domain of Trim37. Trim37 catalyzed the K27/29-linked polyubiquitination of Bcl6, thereby preventing Bcl6 from proteasome-mediated degradation and promoting T_FH_ cell differentiation. Thus, our data demonstrate the essential role of the Trim37-Bcl6 axis in the differentiation of T_FH_ cells that underlies the recurrent respiratory infections observed in patients with Mulibrey nanism.

## Results

### Loss of *Trim37* fails to mount protective humoral immunity against influenza virus

**Fig. 1:**
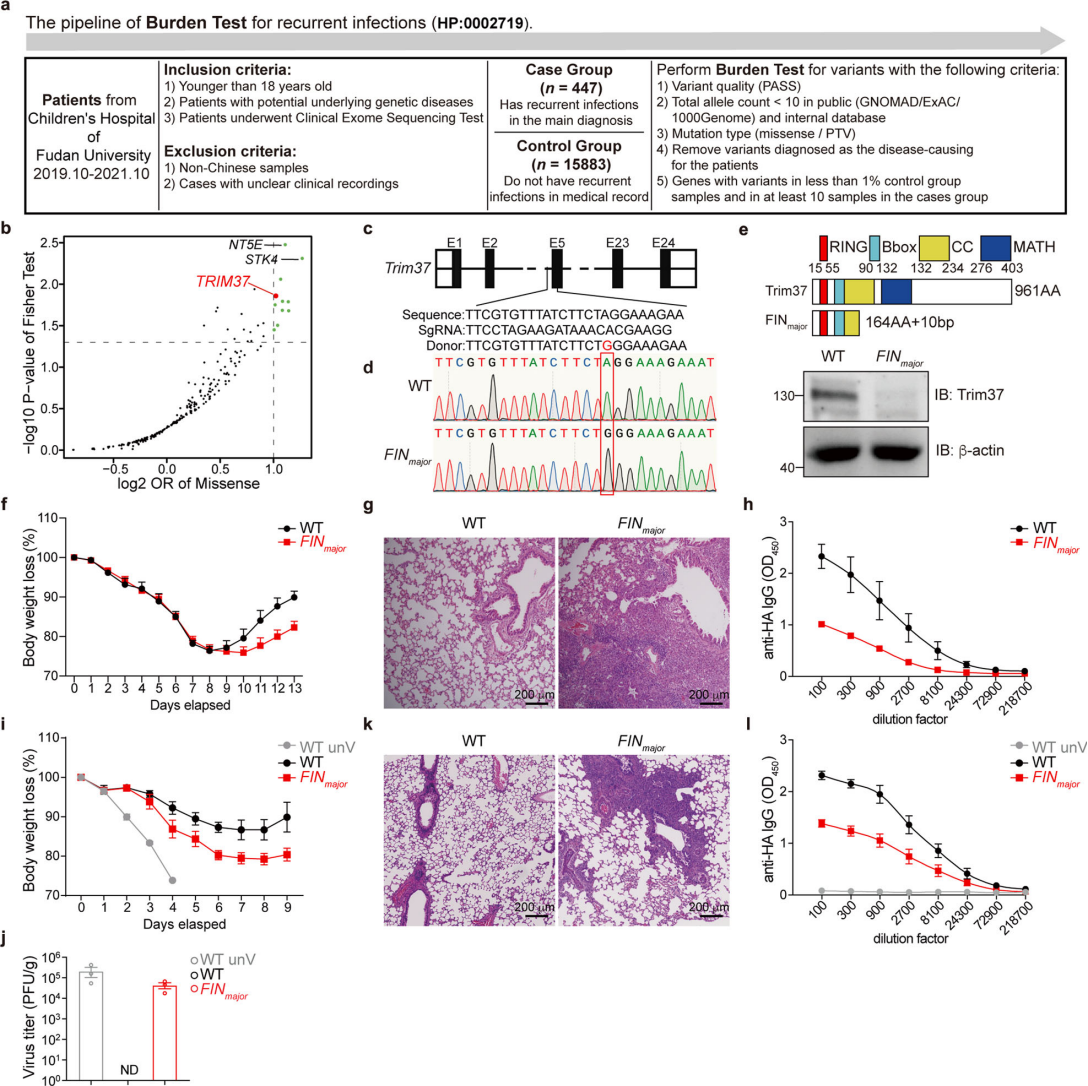
Loss of *Trim37* fails to mount protective humoral immunity against the influenza virus. **a** The pipeline of Burden Test for recurrent infections. **b** Volcano plot of the genetic burden test results for cases with recurrent infections. Points indicate a certain gene with the corresponding log_2_ (OR) and –log_10_ (*P value*) of the Fisher test results. The threshold of OR > 2 and the threshold of *P* < 0.05 are marked by two red dashed lines. Genes above the threshold are shown as green dots, while *TRIM37* is shown as a red dot. **c** The schematic design of *FIN*_*major*_ mice. **d** Sanger sequencing of the WT and *FIN*_*major*_ mice. **e** Schematic presentation of WT Trim37 and *FIN*_*majo*r_ mutant (up) and immunoblot analysis of Trim37 levels in spleen samples obtained from the WT and *FIN*_*major*_ mice (down). **f** The weight loss of WT (*n* = 4) and *FIN*_major_ (*n* = 4) mice following PR8 influenza (0.5 LD_50_) infection. **g** Histopathological analysis was performed by H&E staining 13 days after PR8 influenza (0.5 LD_50_) infection. **h** WT (*n* = 3) and *FIN*_*major*_ (*n* = 3) mice were infected with PR8 influenza (0.5 LD_50_), and the sera were obtained 13 days post-infection. Viral-specific anti-HA IgG in the sera was measured by ELISAs. **i**–**l** WT and *FIN*_*major*_ mice were vaccinated with 10 µg of PR8 vaccine adjuvanted with alum intraperitoneally and boosted with the same agents 2 weeks later. Serum samples were collected 14 days after the second vaccination. These mice were intranasally challenged with a high dose of PR8 influenza virus (10 LD_50_) 19 days after the second vaccination. **i** The weight loss of WT (*n* = 4) and *FIN*_*major*_ (*n* = 4) mice following PR8 influenza (10 LD_50_) infection. The gray line (WT unV, *n* = 2) shows the body weight loss of naive control mice without vaccination. **j** Virus titers of the lungs 4 days post-infection (*n* = 3). **k** Mice were sacrificed on day 9 after influenza virus infection, and lung tissue was collected. Histopathological analysis was performed by H&E staining. **l** Viral-specific anti-HA IgG in the sera was measured by ELISAs (WT, *n* = 4; *FIN*_*major*_, *n* = *5*; WT unV, *n* = 3). Data are representative of one (**b**), two (**i**–**l**) or at least three (**c**–**h**) independent experiments. Data were analyzed by Fisher test (**b**).

A major goal in human genetics is to uncover the associations between natural variants and phenotypic consequences. To understand the relationships between natural variants and recurrent infection, we analyzed the whole-exome sequencing data of patients from the Children’s Hospital of Fudan University. We assessed the association of altering protein-coding variants (protein-truncating variants, PTVs, missense or nonsynonymous variants, MISs) with recurrent infection in 16,330 participants (case group with recurrent infections in the main diagnosis, *n* = 447; control group without recurrent infections in all diagnoses during the hospital period, *n* = 15,883) by performing genetic Burden Test (Fig. 1a). Our screen identified 11 genes (*NT5E, STK4, TBXAS1, TRIM37, SOS1, HPSE2, AFP, SEN4A, RARA, CYP2D6, FKBP10*) carrying 137 variants that have a statistically significant association with the incidence of recurrent infection (*P* < 0.05) (Fig. 1b). Interestingly, *NT5E* and *STK4* deficiency in humans has been reported as associating with combined immunodeficiency and recurrent infections^34–36^.

We also noticed that *TRIM37* mutations in humans have been known to be the genetic cause of the autosomal recessive disorder Mulibrey nanism^30^. Patients with Mulibrey nanism not only have severe growth failure but also suffer from respiratory tract infections in infancy^32,33^. Echoing with these records, our findings also suggested that *TRIM37* is highly associated with an increased incidence of severe infection (Fig. 1b). Considering a case report about antibody deficiency in a girl with Mulibrey nanism^37^, we speculated that TRIM37 might be involved in regulating antibody responses, and recurrent infection in Mulibrey nanism patients might be due to antibody deficiency.

The largest group of Mulibrey nanism patients carry the *FIN*_*major*_ (*c*.493-2A > G) mutation, resulting in aberrant splicing at the next AG site and leading to a 164-aa truncated protein^30^. To explore whether TRIM37 is involved in regulating antibody production and recurrent infection, we constructed mice carrying the *FIN*_*major*_ mutation (Fig. 1c–e). In the *FIN*_*major*_ mice, we did not find significant defects in the development and homeostasis of the adaptive immune system by assaying the percentage of CD4^+^ and CD8^+^ cells in thymuses (Supplementary Fig. S1a) or the percentages of CD4^+^ T cells, CD8^+^ T cells and B cells in peripheral lymph organs (Supplementary Fig. S1b, c), as well as the percentages of naïve CD4^+^ T cells and naïve CD8^+^ T cells in peripheral lymph organs (Supplementary Fig. S1d, e). Hence, *Trim37* deficiency did not seem to impair the development of the adaptive immune system. Consistent with infertility as shown in Mulibrey nanism patients and *Trim37*-deficient mice^38,39^, our in-house developed strains carrying *FIN*_*major*_ variants have shown the infertility phenotype at 8 weeks. Interestingly, after we further infected these mice with the Rico/8/34 (PR8, H1N1) influenza virus (0.5 LD_50_), the *FIN*_*major*_ mice showed more weight loss at 10 days post-infection than the wild-type (WT) mice (Fig. 1f) and had much more severe lung histopathology than the WT mice (Fig. 1g). Highly reminiscent of the respiratory infections in the patients with Mulibrey nanism, these data clearly indicated that *FIN*_*major*_ mice are susceptible to influenza virus infection and of a major defect in the production of anti-influenza IgG (Fig. 1h).

To verify whether the susceptibility to influenza virus infection is due to antibody deficiency, we immunized *FIN*_*major*_ mice with two doses of the PR8 vaccine and challenged these mice with a lethal dose of the PR8 influenza virus (10 LD_50_) 19 days after the second vaccination (Supplementary Fig. S1f). As expected, when compared to the WT mice, the *FIN*_*major*_ mice had greater body weight loss (Fig. 1i), harbored a higher amount of virus (Fig. 1j), and showed more severe damage in the lung (Fig. 1k), indicating insufficient protection that vaccinations may usually confer. Unsurprisingly, the *FIN*_*major*_ mice had a considerably lower level of anti-HA IgG in serum (Fig. 1l).

T_H_1 and cytotoxic CD8^+^ T-cell-mediated cellular immune responses are also important for protective immunity against pathogen invasion. In our study, we did not find a significant difference between IFN-γ^+^CD4^+^ and IFN-γ^+^CD8^+^ cells in the *FIN*_*major*_ mice (Supplementary Fig. S1g–j). Taken together, our data show that the *FIN*_*major*_ variant of *Trim37* leads to poor protective antibody responses upon viral infection or vaccination, which may account for the severe respiratory tract infections often observed with Mulibrey nanism patients.

### Trim37 is required for T_FH_ cell differentiation

**Fig. 2:**
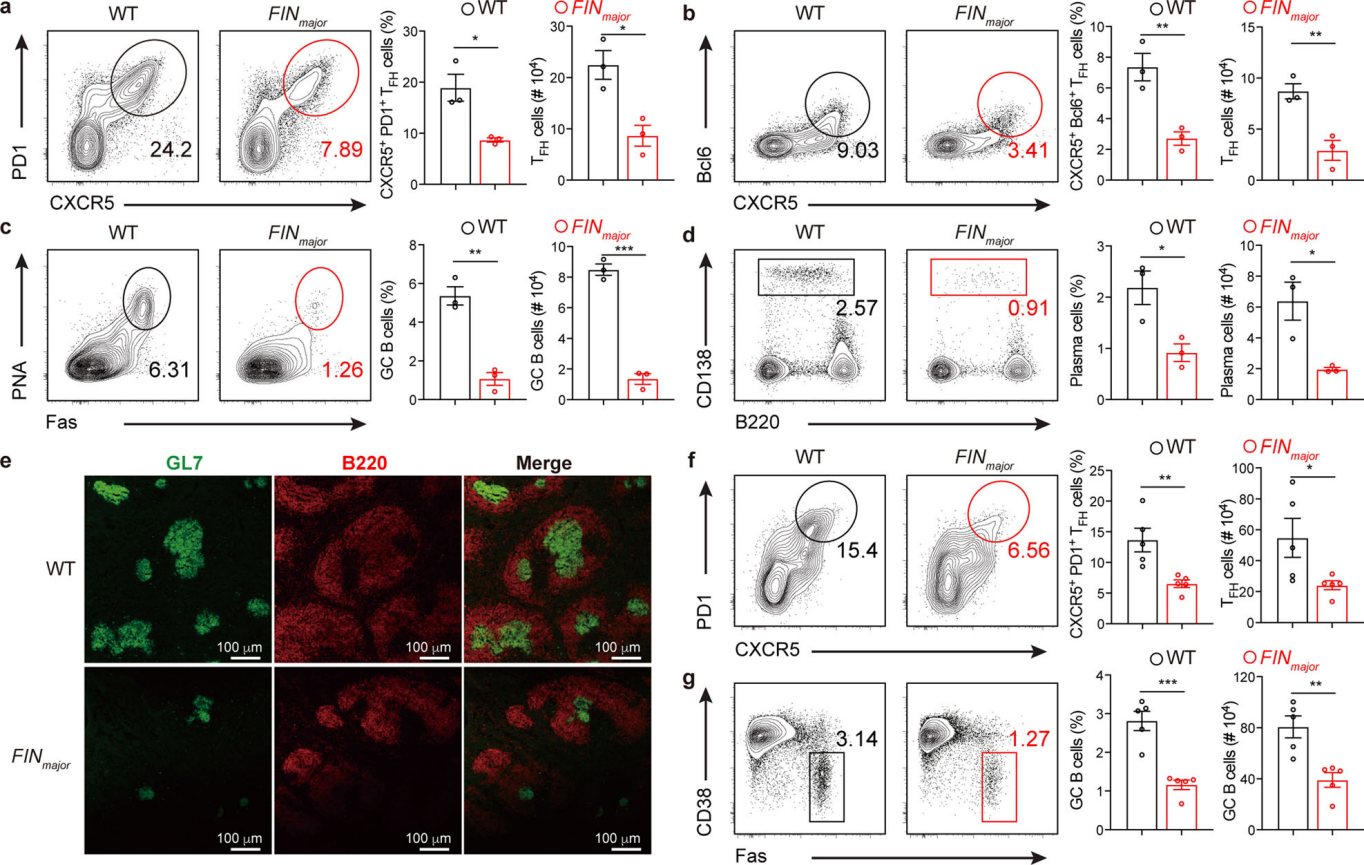
Trim37 is required for T_FH_ cell differentiation. **a**–**e** WT (*n* = 3) and *FIN*_*major*_ (*n* = 3) mice were infected intranasally with PR8 H1N1 influenza virus (0.5 LD_50_). These mice were sacrificed on day 12 after infection. **a** Representative flow cytometry plots illustrate the frequency of CXCR5^+^PD1^+^ T_FH_ cells as a percentage of CD4^+^Foxp3^–^ T cells in the lung draining lymph nodes, and quantification of CXCR5^+^PD1^+^ T_FH_ cells. **b** Representative flow cytometry plots illustrate the frequency of CXCR5^+^Bcl6^+^ T_FH_ cells as a percentage of CD4^+^Foxp3^–^ T cells in the lung-draining lymph nodes, and quantification of CXCR5^+^Bcl6^+^ T_FH_ cells. **c** Representative flow cytometry plots illustrate the frequency of Fas^+^PNA^+^ GC B cells as a percentage of B220^+^ B cells in the lung-draining lymph nodes, and quantification of GC B cells. **d** Representative flow cytometry plots illustrate the frequency of B220^lo^CD138^hi^ plasma cells as a percentage of live cells in the lung-draining lymph nodes, and quantification of plasma cells. **e** Confocal microscopy of the spleen’s germinal center (B220^+^GL7^+^). **f**, **g** WT (*n* = 5) and *FIN*_*major*_ (*n* = 5) mice were vaccinated with 10 µg of PR8 vaccine adjuvanted with alum intraperitoneally. Spleens were collected 10 days after vaccination. **f** Representative flow cytometry plots illustrate the frequency of CXCR5^+^PD1^+^ T_FH_ cells as a percentage of CD4^+^Foxp3^–^ T cells in the spleens and quantification of CXCR5^+^PD1^+^ T_FH_ cells. **g** Representative flow cytometry plots illustrate the frequency of Fas^+^CD38^lo^ GC B cells as a percentage of B220^+^ B cells in the spleens, and quantification of GC B cells. Data are representative of at least three independent experiments and were analyzed by two-tailed unpaired Student’s *t*-test (**a**–**d**, **f**, **g**). Data are mean ± SEM. *P < 0.05, **P < 0.01, ***P < 0.001, ns, not significant.

T_FH_ cells specifically support GC formation and thus play an essential role in antibody responses. To obtain insight into the Trim37-mediated regulation of protective antibody responses upon viral infection or vaccination, we infected the *FIN*_*major*_ mice and their counterpart WT mice with PR8 influenza viruses through an intranasal challenge and analyzed the T_FH_ cells and the antibody responses induced by viral infection. We found that CXCR5^+^PD-1^+^ T_FH_ cells (Fig. 2a; Supplementary Fig. S2a) and CXCR5^+^Bcl6^+^ T_FH_ cells (Fig. 2b; Supplementary Fig. S2b) in the lung-draining lymph nodes and the spleen were largely compromised (~50% loss) in the *FIN*_*major*_ mice as compared to those in the WT mice. Consistently, a considerable decrease in GC B cells (Fig. 2c; Supplementary Fig. S2c), and B220^lo^CD138^hi^ plasma cells (Fig. 2d; Supplementary Fig. S2d) was observed in lung-draining lymph nodes and the spleen. In addition, immunofluorescence staining of B220 and GL7 provided visual images that indicated reduced GC reaction in the spleen of the *FIN*_*major*_ mice (Fig. 2e). Besides, we also observed a spontaneous defect in T_FH_ cell and GC B cell differentiation even in the uninfected *FIN*_*major*_ mice (Supplementary Fig. S2e, f). Thus, these data suggest that Trim37 plays a critical role in T_FH_ cell differentiation and GC formation.

To further pinpoint the critical role of Trim37 in regulating T_FH_ cell differentiation, we immunized WT mice and *FIN*_*major*_ mice with the PR8 vaccine to analyze T_FH_ cell and GC B cell responses. Ten days after immunization, we found that the percentage of CXCR5^+^PD-1^+^ T_FH_ cells was dramatically decreased in the *FIN*_*major*_ mice (Fig. 2f). Moreover, we observed a considerable decrease in B220^+^Fas^+^CD38^lo^ GC B cells (Fig. 2g). These data were highly consistent with our observation that B cells from the *FIN*_*major*_ mice had a diminished ability to produce flu-specific IgG (Fig. 1h). Thus, Trim37 is critical for GC responses and high-affinity antibody production following vaccine immunization. Taken together, these data indicate that Trim37 displays a remarkable impact on the GC response and the production of high-affinity neutralizing antibodies against influenza virus infection and vaccine immunization.

To further confirm the functional effects of Trim37 on T_FH_ cell differentiation, we constructed another *Trim37*^*ko*^ mouse carrying a 7 bp insertion in the exon 4 of *Trim37* (Supplementary Fig. S2a, b). Like in *FIN*_*major*_ mice, we did not find any defects in the development and homeostasis of the adaptive immune system in the *Trim37*^*ko*^ mice (Supplementary Fig. S3c–g). The *Trim37*^*ko*^ mice were also susceptible to influenza virus infection and recovered slowly until 12 days after intranasal challenge (Supplementary Fig. S4a, b), with more severe lung histopathology than the WT mice (Supplementary Fig. S4c). We found that the frequency of CXCR5^+^PD-1^+^ and CXCR5^+^Bcl6^+^ T_FH_ cells in the lung-draining lymph nodes is decreased by over 50% in the *Trim37*^*ko*^ mice as compared to those in the WT mice (Supplementary Fig. S4d, e). Consequently, there was a considerable decrease in GC responses (Supplementary Fig. S4f–h). The *Trim37*^*ko*^ mice also exhibited a significant reduction in the secretion of influenza-specific and neutralizing antibodies, as detected by ELISAs and microneutralizing assays, respectively (Supplementary Fig. S4i, j). Hence, the T_FH_ cell differentiation and GC responses are impaired in both *FIN*_*major*_ and *Trim37*^*ko*^ mice.

### Trim37 promotes T_FH_ cell differentiation in a T-cell-intrinsic manner

**Fig. 3:**
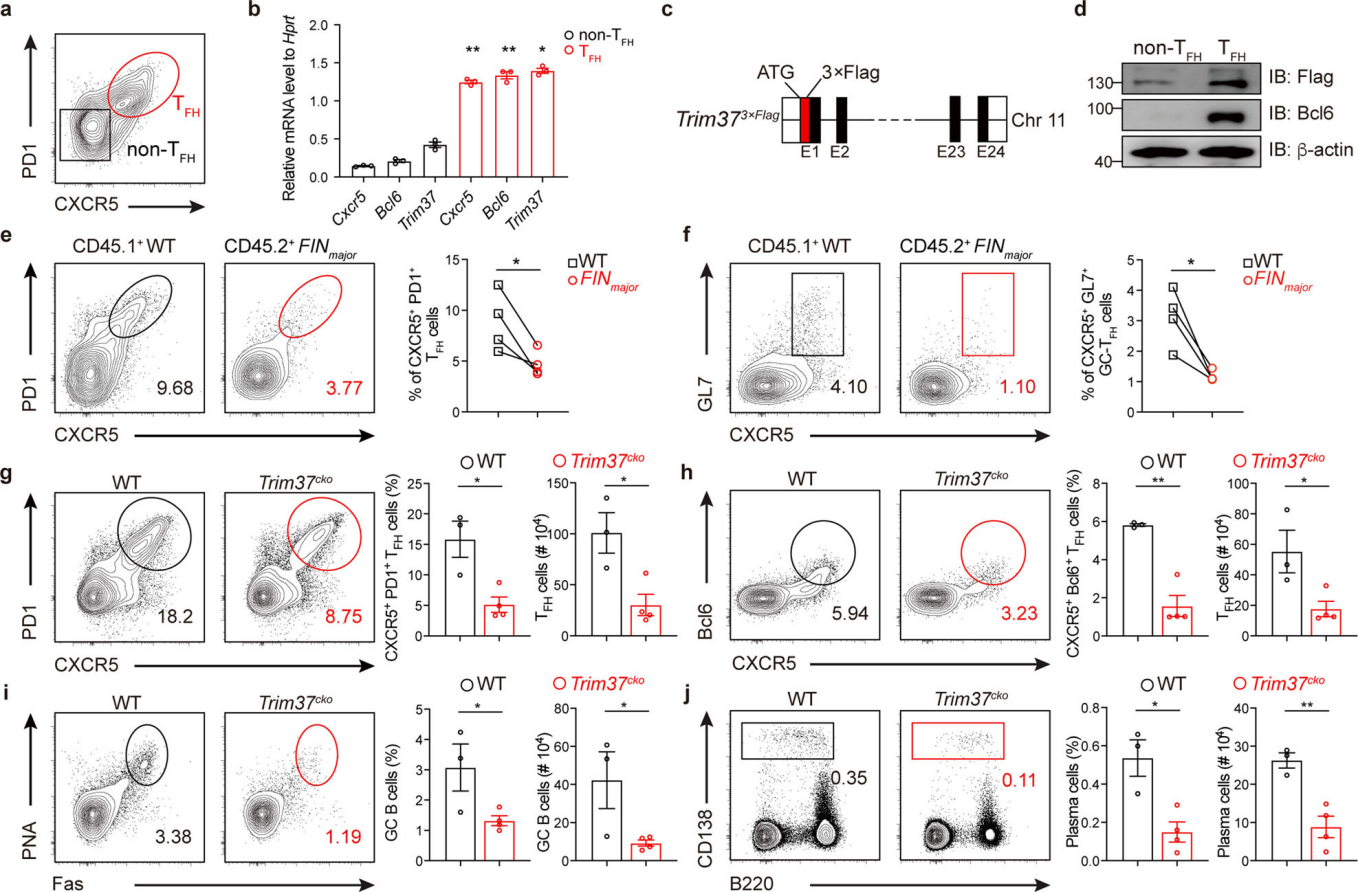
Trim37 promotes T_FH_ cell differentiation in a T-cell-intrinsic manner. **a** Sorting of CXCR5^–^PD1^–^ non-T_FH_ cells and CXCR5^+^PD1^+^ T_FH_ cells from lung-draining lymph nodes and spleens in the C57BL/6 mice infected with PR8 influenza virus. **b** *Cxcr5*, *Bcl6*, and *Trim37* mRNA expressions in non-T_FH_ cells and T_FH_ cells were measured by RT-PCR. **c** Schematic design of the *Trim37*^*3×Flag*^ mice. **d** CXCR5^–^PD1^-^ non-T_FH_ cells and CXCR5^+^PD1^+^ T_FH_ cells were sorted from lung-draining lymph nodes and spleens in the *Trim37*^*3×Flag*^ mice infected with PR8 influenza virus. Flag, Bcl6, and β-actin protein expression was measured by Western blotting. **e**, **f** *FIN*_*major*_ bone marrow chimeric mice were infected intranasally with PR8 H1N1 influenza virus (0.5 LD_50_) and sacrificed on day 12 after infection. Representative flow cytometry plots showing the frequency of CD45.1^+^ (WT, *n* = 4) or CD45.2^+^ (*FIN*_*major*_, *n* = 4) CXCR5^+^ PD1^+^ T_FH_ cells (**e**) and CXCR5^+^ GL7^+^ GC-T_FH_ cells (**f**) as a percentage of CD4^+^ T cells in the spleens. **g**–**j** Flow cytometry analysis of lymphocytes from WT (*n* = 3) and *Trim37*^*cko*^ (*n* = 4) mice at day 10 after infection of PR8 H1N1 influenza virus (0.5 LD_50_). **g**, **h** The frequency and quantification of CXCR5^+^ PD1^+^ (**g**) and CXCR5^+^Bcl6^+^ (**h**) T_FH_ cells as a percentage of CD4^+^ T cells in the draining lymph nodes. **i**, **j** The frequency and quantification of Fas^+^PNA^+^ GC B (**i**) cells and IgD^lo^CD138^hi^ plasma cells (**j**) as a percentage of B220^+^ B cells in the draining lymph nodes. Data are representative of at least three independent experiments, and were analyzed by two-tailed unpaired Student’s *t*-test (**e**–**g**, **j**), or two-way ANOVA (**b**). Data are mean ± SEM. *P < 0.05, **P < 0.01, ***P < 0.001, ns, not significant.

We examined the Trim37 expression level in T_FH_ cells. We infected WT mice with influenza viruses and sorted non-T_FH_ cells (CXCR5^–^PD-1^–^) and T_FH_ cells (CXCR5^+^PD-1^+^) 10 days after PR8 influenza virus infection (Fig. 3a). RT-PCR assays revealed that *Trim37* mRNA was highly expressed in T_FH_ cells (Fig. 3b). Then, we took advantage of *Trim37*^*3×Flag*^ knock-in mice with 3× Flag at the N-terminus of Trim37 to determine Trim37 protein levels in T_FH_ cells (Fig. 3c). We infected the *Trim37*^*3×Flag*^ mice with influenza viruses and sorted non-T_FH_ cells and T_FH_ cells 10 days post-infection. Immunoblot analysis revealed that Trim37 protein levels were also higher in T_FH_ cells than in non-T_FH_ cells (Fig. 3d), suggesting that Trim37 might specifically regulate T_FH_ cell differentiation and function.

We next determined whether Trim37 controls T_FH_ cell differentiation in a T-cell-intrinsic manner. We reconstituted lethally irradiated *Tcrb*^*-/-*^ mice with mixed CD45.2 *FIN*_*major*_ and CD45.1 WT bone marrow (BM) cells at a ratio of 1:1 to generate mixed chimeric mice. Then, these mixed bone marrow chimeric mice were infected with PR8 influenza viruses. T_FH_ cell differentiation was analyzed 12 days post-infection (Supplementary Fig. S5a). Under the same immune environment, CD4^+^ T cells derived from CD45.2^+^ *FIN*_*major*_ BM cells showed an impaired ability to differentiate into T_FH_ cells compared with CD4^+^ T cells derived from CD45.1^+^ WT BM cells (Fig. 3e, f). Likewise, we reconstituted lethally irradiated *Tcrb*^*-/-*^ mice with mixed CD45.2 *Trim37*^*ko*^ and CD45.1 WT BM cells at a ratio of 1:1 to generate mixed chimeric mice (Supplementary Fig. S5b). CD4^+^ T cells derived from CD45.2^+^ *Trim37*^*ko*^ BM cells also showed deficient T_FH_ cell differentiation compared with CD4^+^ T cells derived from WT BM cells 12 days after influenza virus infection (Supplementary Fig. S5c, d). Thus, Trim37 controls T_FH_ cell differentiation in a T-cell-intrinsic manner.

To uncover the specific function of Trim37 in T cells, we generated conditional *Trim37* knockout mice (referred to as *Trim37*^*cko*^) by crossing *Trim37*^*flox*^ mice (Supplementary Fig. S5e, f) with T-cell-specific *Cd4-Cre* mice. Similarly, conditional knockout of *Trim37* in T cells led to significantly decreased T_FH_ differentiation (Fig. 3g, h), GC formation (Fig. 3i), and plasma cell differentiation (Fig. 3j) in the lung draining lymph nodes 12 days after PR8 virus infection. Meanwhile, *Trim37* deficiency seemed not to impair the activation and proliferation of CD4^+^ T cells (Supplementary Fig. S6a, b). In addition, we found that Trim37 did not affect iT_reg_, T_H_1, T_H_2, or T_H_17 cell differentiation in vitro and in vivo (Supplementary Fig. S6c–j). Collectively, these data show that Trim37 governs the GC response by directly regulating T_FH_ cell differentiation.

### Trim37 promotes Bcl6 stability in T_FH_ cell differentiation

**Fig. 4:**
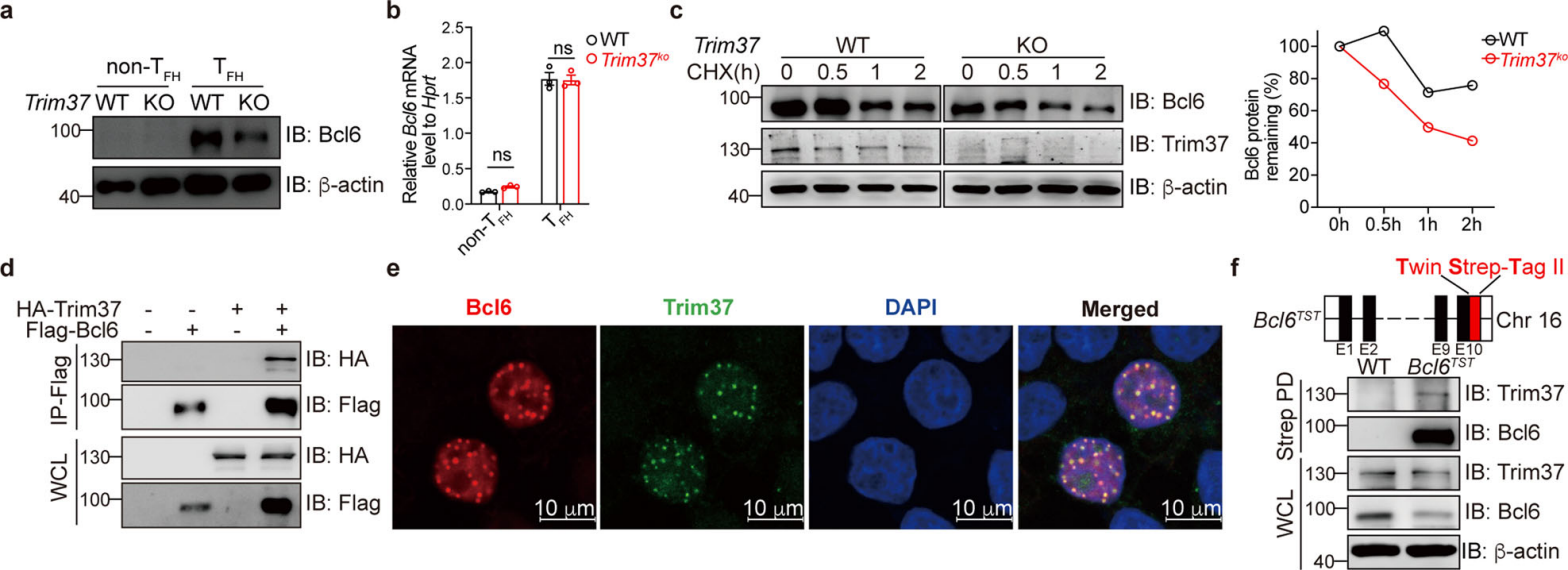
Trim37 binds directly to Bcl6. **a**, **b** CXCR5^–^PD1^–^ non-T_FH_ cells CXCR5^+^PD1^+^ T_FH_ cells were sorted from lung-draining lymph nodes and spleens in the WT and *Trim37*^*ko*^ mice infected with PR8 influenza virus. **a** Bcl6 protein expression was measured by Western blot analysis. **b** *Bcl6* mRNA expression was measured by RT-PCR. **c** The WT and *Trim37*^*ko*^ CD4^+^ T cells were cultured under T_FH-like_ conditions and stimulated with anti-CD3 and anti-ICOS for 4 h. Then, CHX was added to the medium for the indicated hours. Immunoblot analysis of Bcl6 in lysates of CD4^+^ T cells was performed. Residual Bcl6 protein was normalized to actin and presented relative to that before the addition of CHX (right). **d** Immunoblot analysis of lysates from the HEK293T cells transfected with HA-tagged Trim37 and Flag-tagged Bcl6 plasmids, followed by immunoprecipitation with anti-Flag affinity gel and immunoblot analysis with anti-HA or anti-Flag antibodies (Abs). **e** Confocal microscopy of the HEK293T cells transfected with HA-tagged Trim37 and Flag-tagged Bcl6 plasmids. Immunofluorescence was performed with anti-HA (green) and anti-Flag (red) Abs. Nuclei were stained with DAPI (blue). **f** The schematic design of *Bcl6*^*TST*^ mice (up). The WT and *Bcl6*^*TST*^ CD4^+^ T cells were cultured under T_FH-like_ conditions and stimulated with anti-CD3 and anti-ICOS for 4 h, followed by affinity enrichment of TST with Strep-tactin and immunoblot analysis with anti-Bcl6, anti-Trim37 and anti-actin Abs (down). Data are representative of at least three independent experiments, and were analyzed by two-way ANOVA (**b**). Data are mean ± SEM. *P < 0.05, **P < 0.01, ***P < 0.001, ns, not significant.

We then asked how Trim37 regulates T_FH_ cell differentiation. Bcl6 is the master regulator of T_FH_ cell differentiation. We found that the protein levels of Bcl6 were significantly decreased in the *Trim37*^*ko*^ CXCR5^+^PD1^+^ T_FH_ cells (Fig. 4a). However, *Trim37* deficiency did not affect *Bcl6* mRNA levels in T_FH_ cells (Fig. 4b). These results suggested that Bcl6 might be regulated by Trim37 in a post-translational manner. In T cells, anti-CD3 and anti-ICOS stimulation can upregulate Bcl6 expression^17^. After addition of CHX to cultures of CD4^+^ T cells followed by stimulation with anti-CD3 and anti-ICOS antibodies, we observed a significant decrease in the stability of Bcl6 protein of the *Trim37*^*ko*^ CD4^+^ T cells compared to the WT CD4^+^ T cells (Fig. 4c). Moreover, we obtained consistent results when we used flow cytometry to detect the expression of Bcl6 protein (Supplementary Fig. S7a). Thus, Trim37 regulates T_FH_ cell differentiation by promoting Bcl6 stability.

We next investigated how Trim37 regulates Bcl6 expression. Through co-immunoprecipitation assays and confocal experiments, we demonstrated that Trim37 and Bcl6 could directly interact with each other (Fig. 4d, e). Moreover, we investigated whether Trim37 interacted with the Bcl6 protein in T cells. We took advantage of *Bcl6*^*TST*^ knock-in mice with Twin-Strep-Tag (TST) at the C-terminus of Bcl6 (Fig. 4f). CD4^+^ T cells from WT and *Bcl6*^*TST*^ mice were cultured under T_FH-like_ conditions and stimulated with anti-CD3 and anti-ICOS for 4 h, followed by affinity enrichment of TST with Strep-tactin. Immunoblot experiments indicated that Trim37 and Bcl6 could directly interact with each other in T cells (Fig. 4f). Together, these data suggest that Trim37 is associated with Bcl6.

### Trim37-mediated ubiquitination promotes the stability of Bcl6 and differentiation of T_FH_ cells

**Fig. 5:**
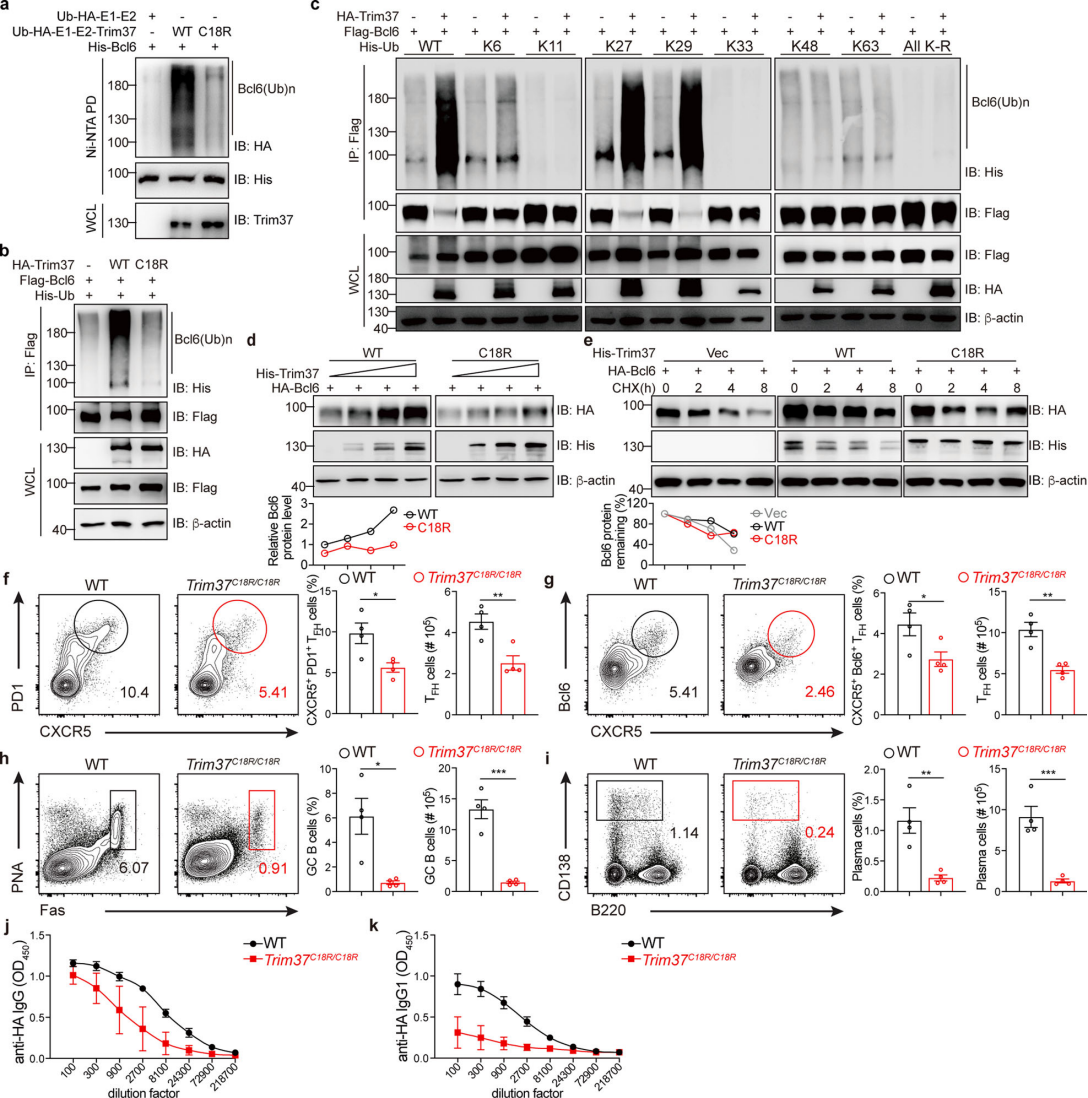
Trim37 targets Bcl6 for K27/29-linked ubiquitination. **a** Reconstituting the *E. coli* ubiquitination system by transforming the pACYC vector that expresses HA-tagged Ub, E1, and E2, with or without Trim37^WT^ or Trim37^C18R^, along with the pET22b-His-Bcl6 vector into BL21 cells. This process was followed by affinity enrichment (Ni-NTA pulldown) of ubiquitinated Bcl6 and immunoblot analysis of Bcl6 expression with anti-His, anti-HA, and anti-Trim37 Abs. **b** Immunoblot analysis of lysates obtained from the HEK293T cells transfected with Flag-tagged Bcl6, His-tagged ubiquitin, and HA-tagged empty vector, Trim37^WT^, or Trim37^C18R^. This process was followed by immunoprecipitation with an anti-Flag affinity gel and analysis with anti-HA, anti-Flag, and anti-His Abs. **c** Immunoblot analysis of lysates obtained from the HEK293T cells transfected with various combinations of plasmids such as HA-tagged Trim37 and Flag-tagged Bcl6 with His-tagged WT-Ub, K6-Ub, K11-Ub, K27-Ub, K29-Ub, K33-Ub, K48-Ub, K63-Ub, and all K-R mutated Ub. Then, assays were performed as in **b**. **d** Immunoblot analysis of Bcl6 in lysates of the HEK293T cells transfected with HA-tagged Bcl6 and increasing doses of His-tagged Trim37^WT^ and Trim37^C18R^ for 24 h. Relative Bcl6 protein, normalized to actin (down). **e** Immunoblot analysis of Bcl6 in lysates obtained from the HEK293T cells transfected with HA-tagged Bcl6 and empty vector, Trim37^WT^, Trim37^C18R^ for 24 h and then treated with CHX for the indicated hours. Residual Bcl6 protein was normalized to actin and presented relative to that before the addition of CHX (down). **f**–**k** WT (*n* = 4) and *Trim37*^*C18R/C18R*^ (*n* = 4) bone marrow chimeric mice were infected with influenza virus for 10 days, and the spleen and serum were collected for the following assays. **f**, **g** Flow cytometry analysis of lymphocytes from WT and *Trim37*^*C18R/C18R*^ bone marrow chimeric mice. The frequency and quantification of CXCR5^+^PD1^+^ (**f**) and CXCR5^+^Bcl6^+^ (**g**) T_FH_ cells as a percentage of CD4^+^ T cells in the draining lymph nodes. **h**, **i** The frequency and quantification of Fas^+^PNA^+^ GC B (**h**) cells and IgD^lo^CD138^hi^ plasma cells (**i**) as a percentage of B220^+^ B cells in the draining lymph nodes. **j**, **k** Viral-specific anti-HA IgG (**j**) and IgG1 (**k**) in the sera were measured by ELISAs. Data are representative of two (**f**–**k**) or at least three (**a**–**e**) independent experiments, and were analyzed by two-tailed unpaired Student’s *t*-test (**f**–**i**). Data are mean ± SEM. *P < 0.05, **P < 0.01, ***P < 0.001, ns, not significant.

Given that Trim37 is an E3 Ub ligase, we assessed whether Trim37 could directly ubiquitinate Bcl6 to regulate T_FH_ cell differentiation. In a reconstituted *Escherichia coli* (*E. coli*) ubiquitination system^40^, we observed a strong ubiquitination signal of Bcl6 when Trim37 and Bcl6 were co-transformed into competent *E. coli* BL21 cells. Bcl6 could be ubiquitinated by WT Trim37 but not the enzymatically inactive mutant (Trim37^C18R^)^23^ (Fig. 5a). Consistently, we also found that Bcl6 was ubiquitinated by WT Trim37 but not Trim37^C18R^ in mammalian cells (Fig. 5b). These findings suggest that Trim37 targets Bcl6 for ubiquitination.

Furthermore, we sought to identify the linkage of the Trim37-mediated ubiquitination of Bcl6 by using the ubiquitin expression plasmids His-Ub-K6, His-Ub-K11, His-Ub-K27, His-Ub-K29, His-Ub-K33, His-Ub-K48, His-Ub-K63 and His-Ub-All K-R (in which all of the lysine residues except K6, K11, K27, K29, K33, K48 or K63, respectively, are replaced). We found that Trim37 mediates K27/29-linked polyubiquitination of Bcl6 (Fig. 5c). All these data suggest that Bcl6 is a direct substrate of Trim37.

To investigate whether Trim37-mediated K27/29-linked polyubiquitination leads to enhanced Bcl6 expression, we co-expressed Bcl6 with Trim37 or E3 ligase dead Trim37^C18R^ mutant in HEK293T cells. The overexpression of WT Trim37, but not Trim37^C18R^ mutant up-regulated the level of Bcl6 protein in HEK293T cells (Fig. 5d). We also introduced the protein synthesis inhibitor cycloheximide (CHX) to treat HEK293T cells that overexpressed WT Trim37 or Trim37^C18R^. Immunoblot experiments indicated that WT Trim37 but not Trim37^C18R^ prolonged the stability of Bcl6 (Fig. 5e). These data suggest that Trim37 could promote the stability of Bcl6 via its E3 ligase activity.

To determine whether Trim37 controls T_FH_ cell differentiation through its ubiquitin E3 ligase activity in vivo, we generated *Trim37*^*C18R*^ knock-in mice (Supplementary Fig. S7b, c). Then, we reconstituted lethally irradiated *Tcrb*^*-/-*^ mice with *Trim37*^*C18R/C18R*^ or WT BM cells to generate bone marrow chimeric mice and challenged these chimeric mice with PR8 influenza virus (Supplementary Fig. S7d). On day 10 post-infection, the *Trim37*^*C18R/C18R*^ bone marrow chimeric mice showed fewer T_FH_ cells than the WT bone marrow chimeric mice (Fig. 5f, g). Consistent with this observation, the *Trim37*^*C18R/C18R*^ bone marrow chimeric mice had dramatically decreased GC B cells (Fig. 5h) and plasma cells (Fig. 5i). We also found that the *Trim37* ^*C18R/C18R*^ chimeric mice had a diminished ability to produce HA-specific IgG and IgG1, as shown by ELISA (Fig. 5j, k). All these data demonstrate that the E3 ligase activity of Trim37 is essential to control T_FH_ differentiation, by promoting the stability of Bcl6.

### Trim37 ubiquitinates Bcl6 at the K227, K302, K327, K535, and K689 residues

**Fig. 6:**
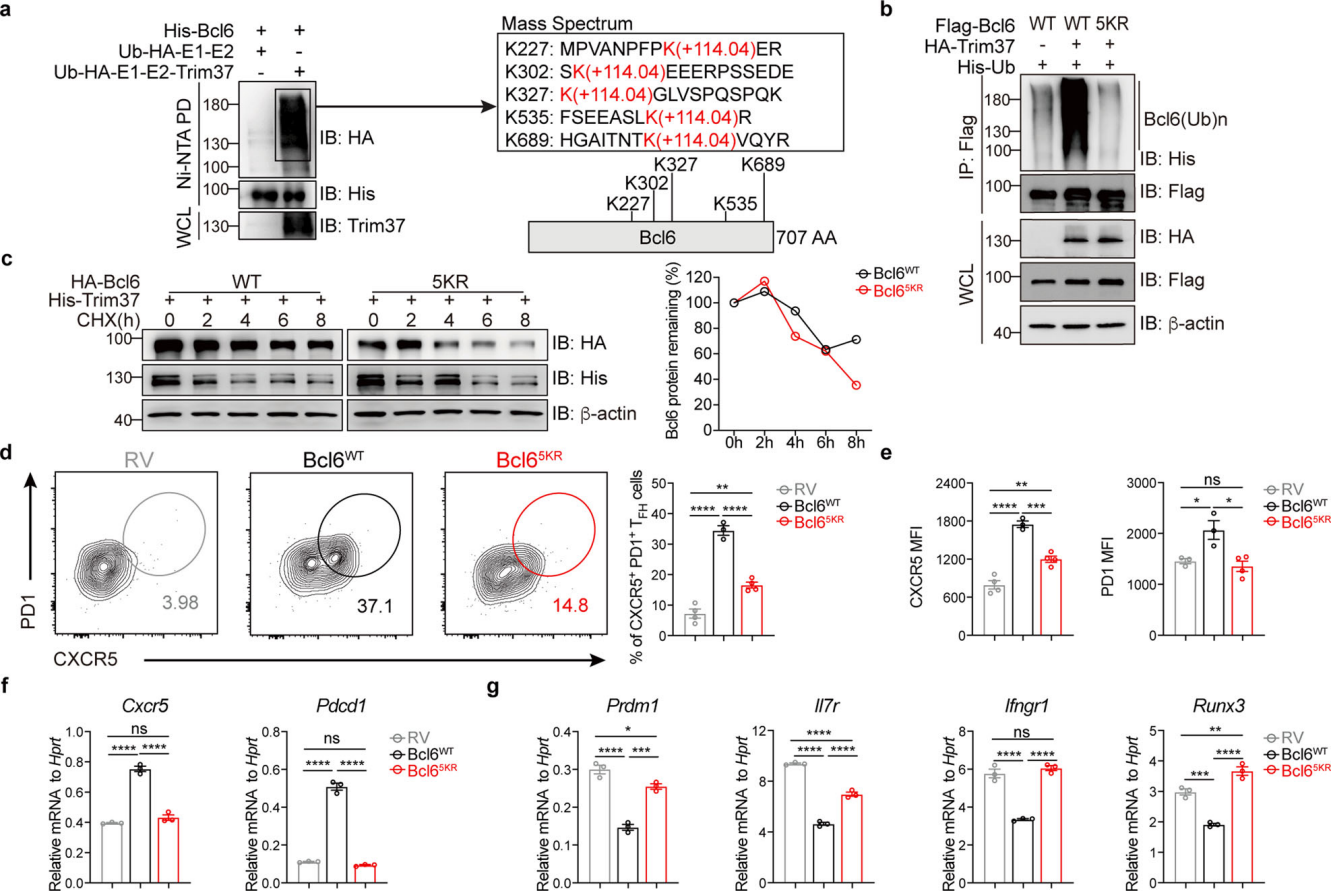
Trim37 ubiquitinates Bcl6 at the K227, K302, K327, K535, and K689 residues. **a** Reconstituting the *E. coli* ubiquitination system by transforming the pACYC vector that expresses HA-tagged Ub, E1, and E2, with or without Trim37^WT^, along with the pET22b-His-Bcl6 vector into BL21 cells. This step was followed by two-step affinity enrichment. The supernatant containing ubiquitinated Bcl6 was analyzed by mass spectrometry. Site mapping for ubiquitination of Bcl6 is shown (right). **b** Immunoblot analysis of lysates obtained from the HEK293T cells transfected with Flag-tagged Bcl6^WT^ or Bcl6^5KR^ and plasmids as indicated. This step was followed by immunoprecipitation with an anti-Flag affinity gel and analysis with anti-HA, anti-Flag, and anti-His Abs. **c** HEK293T cells were transfected with HA-tagged Bcl6^WT^ or Bcl6^5KR^ and Trim37^WT^. Then, the cells were treated with CHX for the indicated hours. Immunoblot analysis of Bcl6 in lysates was performed. Residual Bcl6 protein was normalized to actin and presented relative to that before the addition of CHX (right). **d** OT-II *Bcl6*-deficient CD4^+^ T cells transduced with RV, Bcl6^WT,^ or Bcl6^5KR^ retroviruses, and then transfer to WT mice (RV, *n* = 4, Bcl6^WT^, *n* = 3, Bcl6^5KR^, *n* = 4). Representative flow cytometry plots showing the frequency of CXCR5^+^PD1^+^ T_FH_ cells as a percentage of CD4^+^GFP^+^ T cells in the spleen on day 7 after NP-OVA/alum immunization. **e** The median fluorescence index (MFI) of CXCR5 and PD1 in GFP^+^ OT-II T cells transduced with RV (*n* = 4), Bcl6^WT^ (*n* = 3), or Bcl6^5KR^ (*n* = 4) retroviruses. **f** The mRNA expression of *Cxcr5* and *Pdcd1* was detected in T_H_0 cells transduced with RV, Bcl6^WT^, or Bcl6^5KR^ retroviruses. **g** The mRNA expression of *Prdm1*, *Il7r*, *Ifngr1* and *Runx3* was detected in T_H_0 cells transduced with RV, Bcl6^WT,^ or Bcl6^5KR^ retroviruses. Data are representative of one (**a**) or at least three (**b**–**g**) independent experiments, and were analyzed by One-way ANOVA (**d**–**g**). Data are mean ± SEM. *P < 0.05, **P < 0.01, ***P < 0.001, ****P < 0.0001, ns, not significant.

A previous study demonstrated that canonical ubiquitination sites in proteins bore Gly-Gly adducts to the side chain of lysine (K) residues^40^. Gly-Gly adducts were found on five lysines (K227, K302, K327, K535, K689) in Bcl6, as revealed by mass spectrum analysis (Fig. 6a). Then, we simultaneously replaced all 5 Bcl6 lysine residues with arginine (5KR) and co-expressed ubiquitin, Trim37, and the WT (Bcl6^WT^) or 5KR mutant (Bcl6^5KR^) of Bcl6 in HEK293T cells. The in vivo ubiquitination assay showed that the ubiquitination of the Bcl6^5KR^ was significantly attenuated compared to that of the Bcl6^WT^ (Fig. 6b). In addition, the Bcl6^5KR^ displayed decreased stability in the presence of Trim37 (Fig. 6c).

Moreover, we investigated whether this mutant Bcl6 can influence the function of Bcl6 in vivo. To test this hypothesis, we transduced *Bcl6*-deficient OT-II CD4^+^ T cells with retroviruses expressing GFP alone (RV-GFP), WT Bcl6 (Bcl6^WT^-GFP), or 5KR mutant Bcl6 (Bcl6^5KR^-GFP), transferred these cells into B6 WT mice and analyzed T_FH_ cell differentiation 7 days after NP-OVA plus alum immunization (Supplementary Fig. S8a). Consistently, we observed a sharp decrease in T_FH_ cell differentiation in the OT-II cells with Bcl6^5KR^-GFP compared to the OT-II cells with Bcl6^WT^-GFP (Fig. 6d). Compared to the *Bcl6* mRNA level, we observed a slightly low expression of Bcl6 protein in Bcl6^5KR^-GFP^+^ T cells before adoptive transfer (Supplementary Fig. S8b, c). We investigated whether this mutant Bcl6 can influence the function of Bcl6. Compared to the overexpression of Bcl6^WT^ in CD4^+^ T cells, overexpression of Bcl6^5KR^ barely induces the expression of CXCR5 and PD-1 (Fig. 6e, f). In contrast, overexpression of Bcl6^WT^ in CD4^+^ T cells represses *Prdm1*, *Il7r*, *Ifngr1*, *Runx3*, *Gata3*, *Klf2*, *Ccr7* and *S1pr1*, while overexpression of Bcl6^5KR^ in CD4^+^ T cells barely represses expression of these genes (Fig. 6g; Supplementary Fig. S8d). All these data indicated that Trim37-mediated non-proteolytic ubiquitination of Bcl6 at the 5 lysine residues, is critical for the stability of Bcl6 and significantly impacts the transcription of its target genes in T cells.

### Trim37 promotes the stability of Bcl6 and differentiation of T_FH_ cells dependent on the MATH domain

**Fig. 7:**
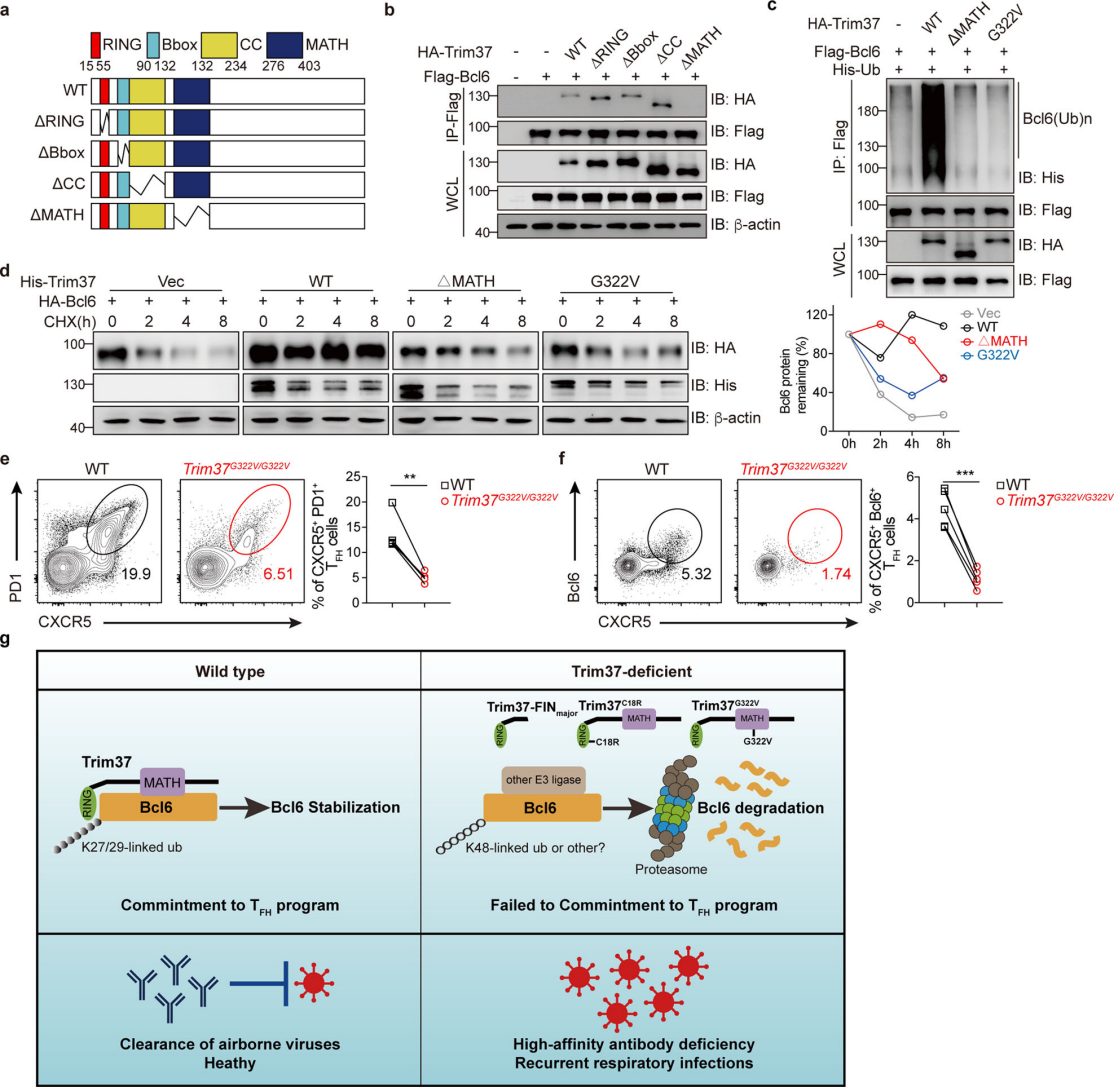
Trim37 promotes the stability of Bcl6 and differentiation of T_FH_ cell dependent on the MATH domain. **a** Schematic presentation of full-length Trim37 and its mutants. RING, ring-finger domain; Bbox, B-box domain; CC, coiled-coil domain; MATH, meprin and TRAF–C homology domain. **b** Immunoblot analysis of lysates obtained from the HEK293T cells transfected with Flag-tagged Bcl6 and HA-tagged full-length Trim37 or Trim37 mutants as indicated, followed by immunoprecipitation with anti-Flag affinity gel and immunoblot analysis with anti-HA or anti-Flag Abs. **c** Immunoblot analysis of lysates obtained from the HEK293T cells transfected with Flag-tagged Bcl6, His-tagged ubiquitin, and the HA-tagged vectors Trim37^WT^, Trim37^△MATH^, Trim37^G322V^. This process was followed by immunoprecipitation with an anti-Flag affinity gel and analysis with anti-HA, anti-Flag, and anti-His Abs. **d** Immunoblot analysis of Bcl6 in lysates of the HEK293T cells transfected with HA-tagged Bcl6 and empty vector, Trim37^WT^, Trim37^△MATH^, and Trim37^G322V^. Then, the cells were treated with CHX for the indicated hours. Residual Bcl6 protein was normalized to actin and presented relative to that before the addition of CHX (right). **e**, **f** WT and *Trim37*^*G322V/G322V*^ bone marrow chimeric mice were infected with the influenza virus for 10 days, and the spleens were collected for FACS analysis. **e** Representative flow cytometry plots showing the frequency of CD45.1^+^ (WT, *n* = 4) or CD45.2^+^ (*Trim37*^*G322V/G322V*^, *n* = 4) CXCR5^+^PD1^+^ T_FH_ cells as a percentage of CD4^+^ T cells in the spleen. **f** Representative flow cytometry plots showing the frequency of CD45.1^+^ (WT, *n* = 4) or CD45.2^+^ (*Trim37*^*G322V/G322V*^, *n* = 4) CXCR5^+^Bcl6^+^ T_FH_ cells as a percentage of CD4^+^Foxp3^-^ T cells in the spleen. **g** Schematic model for the Trim37-mediated ubiquitination of Bcl6 and regulation of T_FH_ cell differentiation. Data are representative of two (**e**, **f**) or at least three (**a**–**d**) independent experiments, and were analyzed by two-tailed paired Student’s *t*-test (**e**, **f**). Data are mean ± SEM. *P < 0.05, **P < 0.01, ***P < 0.001, ns, not significant.

Trim37 contains the MATH domain on its C-terminal following the RBCC (RING-B-box-coiled-coil) domain, which is predicted to mediate protein–protein interactions^41^. To explore whether the MATH domain governed the interaction between Trim37 and Bcl6, we performed coimmunoprecipitation experiments by overexpressing full-length Trim37 or various Trim37 truncations with Bcl6 (Fig. 7a). The results showed that full-length Trim37 and truncated Trim37 mutants, except the MATH domain-deleted mutant, were able to immunoprecipitate with Bcl6 (Fig. 7b). These data indicated that Trim37 interacts with Bcl6 via its MATH domain.

To investigate whether the Trim37–Bcl6 interaction is required for Bcl6 ubiquitination, we co-transfected HA-Trim37^△MATH^, Flag-Bcl6, and His-tagged ubiquitin and harvested cells 28 h after transfection. Expression of the MATH domain-deleted Trim37 with Bcl6 did not result in its ubiquitination (Fig. 7c). A c.965G > T (p.G322V) mutation located in the MATH domain of TRIM37 was described in Mulibrey nanism patients^42^. Interestingly, this G322V mutation (Trim37^G322V^) abolished the ability of Trim37 to ubiquitinate Bcl6 (Fig. 7c). These data indicated that Trim37 ubiquitinates Bcl6 in a MATH domain-dependent manner.

To further confirm whether the Trim37–Bcl6 interaction is required for the stability of Bcl6, we also introduced CHX to treat HEK293T cells that overexpressed WT Trim37, Trim37^△MATH^ or Trim37^G322V^. Immunoblot experiments indicated that expression of MATH domain-deleted and G322V mutant Trim37 led to the instability of Bcl6 (Fig. 7d). These data suggest that Trim37–Bcl6 interaction is essential for promoting the stability of Bcl6.

To explore the functional consequence of the G322V mutation in vivo, we constructed mice carrying the *Trim37*^*G322V*^ mutation (Supplementary Fig. S9a, b) and generated *Trim37*^*G322V/G322V*^ mixed bone marrow chimeric mice (Supplementary Fig. S9c). Ten weeks after bone marrow reconstitution, we infected these chimeric mice with the PR8 influenza virus. As shown in (Fig. 7e, f), the *Trim37*^*G322V/G322V*^ CD4^+^ T cells had impaired T_FH_ cell differentiation, compared to the WT CD4^+^ T cells. Altogether, these data demonstrated that Trim37 promotes the stability of Bcl6 dependently on its MATH domain, and the G322V mutation disrupts the function of Trim37 in promoting T_FH_ cell differentiation.

## Discussion

Here we establish the causality between the E3 Ub ligase TRIM37 and severe respiratory infection (Fig. 7g). Through a genetic burden test, we identified *TRIM37* variants significantly associated with recurrent infection. By constructing multiple *Trim37* mutant mice (*FIN*_*major*_, *Trim37*^*ko*^, *Trim37*^*cko*^, *Trim37*^*C18R/C18R*^, *Trim37*^*G322V/G322V*^), we found that respiratory tract infections and antibody deficiency in Mulibrey nanism patients could be ascribed to TRIM37-mediated regulation of protective antibody responses. Trim37-mediated ubiquitination prolonged the stability of Bcl6 and subsequently promoted differentiation of T_FH_ cells. Thus, our findings suggest a previously unknown Trim37-Bcl6 axis that regulates T_FH_ cell differentiation and high-affinity antibody production.

*TRIM37* mutations found in humans cause a rare autosomal recessive disorder characterized by severe prenatal-onset growth failure, infertility, cardiomyopathy, fatty liver, type 2 diabetes, and tumorigenesis named Mulibrey nanism^30^. A clinical review of 85 *FIN*_*major*_ patients’ hospital records revealed that these patients suffer from respiratory tract infections, co-incident with an increased mortality rate^32,33^. All this information indicated the importance of TRIM37 in protective immunity against infection. In light of a previous case report about antibody deficiency in a girl with Mulibrey nanism^37^, TRIM37 might play a critical role in the control of T_FH_ cell differentiation, germinal center formation, and antibody production. Our study then took advantage of genetic mouse models to address the exact cause of susceptibility to infection in patients with Mulibrey nanism. We found that Trim37 exerts a remarkable impact on the T_FH_ cell response and the production of neutralizing antibodies against influenza infection in the *Trim37* mutant mice. Moreover, the *FIN*_*major*_ mice have poor protective humoral immunity followed by influenza vaccine immunization, resulting in susceptibility to influenza virus infection. Our results show that respiratory tract infections and antibody deficiency in Mulibrey nanism patients could be ascribed to deficient T_FH_ cell and germinal center B cell responses.

We found that Trim37 may regulate Bcl6 levels in a post-transcriptional mechanism. Indeed, Trim37 directly interacts with Bcl6 and targets Bcl6 predominantly for K27/29-linked ubiquitination. Trim37 prolongs the stability of Bcl6 dependent on its E3 Ub ligase activity, while *Trim37* mutant mice carrying the enzymatically inactive Trim37^C18R^ display a diminished ability to differentiate into T_FH_ cells and an insufficient production of anti-influenza high-affinity antibodies. All these findings demonstrated that Trim37 is the direct E3 Ub ligase of Bcl6 and that Trim37 promotes T_FH_ cell differentiation by ubiquitinating and stabilizing Bcl6. Moreover, Trim37 mediates non-proteolytic ubiquitination of Bcl6 at 5 key lysines (K227, K302, K327, K535, and K689) residues. This 5KR mutant Bcl6 showed an abrogated ubiquitination level, resulting in decreased stability of Bcl6 and diminished T_FH_ cell differentiation. Trim37 contains the MATH domain following the RBCC domain that is predicted to mediate protein–protein interactions^41^. As expected, Trim37 interacts with and ubiquitinates Bcl6 in a manner dependent on its MATH domain. Interestingly, the c.965 G > T (p.G322V) mutation located in the MATH domain of TRIM37 was described in Mulibrey nanism patients^42^. This G322V mutation can disrupt the Bcl6 ubiquitination by Trim37 and consequently dampen T_FH_ cell differentiation. Taken together, these data established that the Trim37–Bcl6 interaction was essential for promoting the stability of Bcl6 and the differentiation of T_FH_ cells. Furthermore, we generated *Trim37*^*bko*^ mice by crossing *Trim37*^*fl/fl*^ mice with *Cd19-cre* knock-in mice, and the preliminary study suggests that Trim37 can also directly regulate the differentiation of germinal center B cell differentiation (Data not shown). How Trim37 regulates the function of germinal center B cells and whether it regulates germinal center B cell differentiation by regulating Bcl6 ubiquitination remain for further research.

Moreover, post-transcriptional regulation also affects BCL6 protein levels in B lymphocytes^43^. For example, phosphorylation of BCL6 by MAPK results in BCL6 degradation^44^, and FBXO11 mediates proteasome-mediated degradation of BCL6 in B lymphocytes^45^. In contrast, PELI1 induces K63-linked BCL6 polyubiquitination and promotes BCL6 stabilization^46^, and AIP inhibits BCL6 degradation by regulating the deubiquitinase UCHL1^47^. In addition, small molecule BI-3802 has been investigated to induce the degradation of BCL6 protein through E3 ubiquitin ligase SIAH1^48,49^. All these researches indicate the complex ubiquitin modification of the BCL6 protein. The linkage of E3 ligase-mediated ubiquitination of substrates determines the biological consequence. K11 and K48-linked polyubiquitination targets the substrate to the proteasome for degradation, while other atypical ubiquitin modifications (M1, K6, K27, K29, K33, or K63) may lead to nonprotelytic consequences^50^. In this work, we identified that Trim37 targets Bcl6 predominantly for K27/29 linked ubiquitination in T_FH_ cells, thereby promoting the stability of the Bcl6 protein. However, whether other E3 ubiquitin ligases are involved in the post-transcriptional modification of the Bcl6 protein, especially the E3 ubiquitin ligase responsible for inducing the degradation of Bcl6 in T_FH_ cells, needs further study.

A previous study has reported that specific *TRIM37* mutations were associated with a selective impairment in both frequency and proliferative ability of the CD4^+^ T cell subset, along with a terminally differentiated memory phenotype in both CD4^+^ T cells and CD8^+^ T cells^51^. However, *Trim37* mutant mice in our study did not exhibit these phenotypes. It is likely that the specific *TRIM37* variants present in this case encoded mutated TRIM37 protein, altering its functions in T cells. Another possibility is that our inbred mice are maintained in specific pathogen-free barrier animal facilities, while the diverse human living environment, especially the presence of pathogens, is also important to modulate gene effects^52^.

In summary, we identified Trim37 as the key E3 Ub ligase for Bcl6 in T cells. We have demonstrated that the Trim37-Bcl6 axis is critical for T_FH_ cell differentiation and antibody production. The defective T_FH_ cell responses may actually account for the defect in protective humoral immunity observed with *Trim37* mutant mice and patients who harbor the disease-causing *TRIM37* variants. Therapeutic restoring the functionality of the TRIM37 mutant may provide a novel avenue to battle against recurrent infection and treat Mulibrey nanism.

## Materials and methods

### Genetic burden test and participants

Clinical exome sequencing data of patients from the Children’s Hospital of Fudan University with potential underlying genetic disorders between October 1st, 2019, and September 30th, 2021, were collected. The patients were divided into the RI (recurrent infections) and non-RI groups based on the presence or absence of recurrent infections in the primary diagnosis. The genetic burden test was performed as follows: (1) Each variant was annotated to obtain its mutation type, which was further grouped into four types (protein-truncating variants (PTVs), missense or nonsynonymous variants (MISs), synonymous variants, and noncoding variants) according to the transfer table shown in Supplementary Table S1. (2) For each gene in each sample, the number of variants with each mutation type was calculated and summarized. (3) We applied Fisher’s exact test to determine whether the number of variants of each mutation type in each gene was significantly higher in the RI group than in the non-RI group (Supplementary Table S2). In particular, tests of PTV and MIS variants were used to identify RI-related burden genes. Synonymous variants and noncoding variants were treated as the near-neutral background, and genes with significant differences found at the synonymous or noncoding level were filtered out. The threshold of significance was *P* < 0.05 and OR > 2. The criteria for genetic testing were approved by the ethics committees of Children’s Hospital, Fudan University (2015-130). Written informed consent was signed by at least one of the patient’s parents.

### Mice

Mice were bred and maintained in specific pathogen-free barrier animal facilities. All mice were used according to protocols approved by the Institutional Animal Care and Use Committee of Shanghai Institute of Biochemistry and Cell Biology. C57BL/6N mice were purchased from Shanghai Laboratory Animal Company. *Tcrb* ^*-/-*^ mice were purchased from the Model Animal Research Center of Nanjing University. CD45.1 congenic mice and *Cd4-cre* transgenic mice have been described previously^53^. OT-II and *Bcl6*^*fl/fl*^ mice were purchased from the Jackson Laboratories. *Bcl6*^*fl/fl*^ mice were bred with *Cd4-cre* and OT-II mice to generate OT-II *Cd4-cre*^*+/-*^ *Bcl6*^*fl/fl*^ mice. Mice at 6–8 weeks were used for cell culture, immunization, and infection analyses. All mice were age- and sex-matched.

### Generation of *Trim37* mutant mouse models through zygote microinjection

*FIN*_*major*_, *Trim37*^*ko*^, and *Trim37*^*C18R*^ mutant mice were generated by zygote microinjection as described in a previous report^54^. In brief, the mixture of *Cas9* mRNA (100 ng/μL), sgRNA (100 ng/μL), and oligo donor (50 ng/μL) (without donor for the *Trim37*^*ko*^ model) was diluted in RNase-free water, centrifuged at 4 °C and 13,200 rpm for 10 min and then injected into the cytoplasm of zygotes harvested from C57BL/6N females (mated with C57BL/6N males) using a micromanipulator and a FemtoJet microinjector (Eppendorf). The embryos were cultured in KSOM medium until the two-cell stage and then transplanted into the oviducts of 0.5-day post-coitum (dpc) pseudopregnant ICR females. F0 mosaic mice carrying expected genotypes were selected by Sanger sequencing of PCR products and then backcrossed with WT mice for 3–4 generations to obtain heterozygous mice. All sequence information for sgRNAs and primers is shown in Supplementary Table S3.

### Construction of knock-in mice through semicloning-technology

*Trim37*^*3×Flag*^, *Trim37*^*flox*^, *Trim37*^*G322V*^, and *Bcl6*^*TST*^ knock-in mice were constructed by semi-cloning technology combined with CRISPR-Cas9 as in previous works^55,56^. Briefly, sgRNA oligos for *Trim37*^*3×Flag*^, *Trim37*^*flox*^, *Trim37*^*G322V*^, and *Bcl6*^*TST*^ were synthesized and ligated into the Px330-mCherry plasmid (Addgene #98750), which expressed Cas9 and sgRNA for each edited site. Donors for *Trim37*^*3×Flag*^, *Trim37*^*G322V*^, and *Bcl6*^*TST*^ were cloned into pMD19T vector, named 19T-HD-*Trim37*^*3×Flag*^, 19T-HD-*Trim37*^*G322V*^, and 19T-HD-*Bcl6*^*TST*^, respectively. Two donors for *Trim37*^*flox*^ directly synthesized 98 bp oligos, including the flox sequence shown in Supplementary Fig. S5. Px330-mCherry plasmid and donor were transfected into androgenetic haploid embryonic stem cells (AG-haESCs or O48)^56^, which were cultured in DMEM with 15% FBS (Excell Bio), penicillin-streptomycin, nonessential amino acids, NUC, L-glutamine, 2-mercaptoethanol, 1000 U mL^−1^ Lif, 1 μM PD03259010 (Selleck) and 3 μM CHIR99021 (Selleck). The mCherry-positive haploid cells were enriched through FACS and plated in one well of the 6-well plate for single-cell expansion. Six to 7 days after plating, single-cell clones were picked and separated into two parts, one for passaging and the other for sequencing to determine the knock-in genotype. For the generation of semi-cloning mice, *Trim37-*modified O48 cells were treated with 0.05 μg/mL demecolcine solution (Sigma) for 10–12 h and synchronized to M phase for the ICAHCI (intracytoplasmic AG-haESC injection) experiment. The reconstructed two-cell embryos were also transplanted into the oviducts of 0.5 dpc pseudopregnant ICR females and born after 19 days. Then, the *Trim37*-modified F0 heterozygous mice were backcrossed with C57BL/6N males for 3–4 generations. All sequence information for sgRNAs and primers is shown in Supplementary Table S3.

### Infection with influenza virus and immunization

The WT A/Puerto Rico/8/34 H1N1 (PR8) influenza virus was obtained by cotransformation of 8 plasmids through a reverse genetic system^57^. The virus was expanded using Madin-Darby canine kidney (MDCK) cells, and the titer was measured. The expanded virus fraction was used after loading, freezing, and storage at –80 °C. Before use, the viruses were melted on ice and diluted with 1× PBS filtered through a 0.22-μm filter. Eight-week-old mice were infected with the influenza virus at a dose of 0.5 LD_50_ (15 PFU) or 10 LD_50_ (300 PFU) per 30 μL. PR8 influenza virus vaccine was provided by Dr. Ze Chen. Mice were vaccinated with 10 μg of PR8 vaccine with an intraperitoneal alum adjuvant (Thermo Fisher Scientific) and boosted with the same agents 2 weeks later. Serum samples were collected 2 weeks after the second vaccination. These mice were intranasally challenged with a high dose of PR8 influenza virus (10 LD_50_) 19 days after the second vaccination.

### Flow cytometry

The procedures used in this study were previously described^53^. The antibodies for surface markers used in flow cytometry were as follows: anti-CD4 (GK1.5), anti-CD62L (MEL14), anti-GL7 (GL7), anti-B220 (RA3-6B2), anti-Fas (Jo2), streptavidin-PE, streptavidin-BV421, anti-CD45.1 (A20), anti-CD45.2 (104), anti-CD38 (90/CD38), Fixable Viability Dye 510, Fixable Viability Dye 780, and 7-AAD from BD Pharmingen; anti-CD44 (IM7), biotinylated anti-CXCR5 (SPRCC5), anti-IgM (II/41), and Fc Blocker (clone 93) from Invitrogen; anti-CD25 (PC61), anti-PD1 (29F.1A12), anti-IgD (11–26 c.2a), anti-IgG1 (RMG1-1), and anti-CD138 (281-2) from Biolegend; and PNA (FITC) from Vector. The TF antibodies for flow cytometry were as follows: anti-Foxp3 (JFK-16s) from Invitrogen and anti-Bcl6 (K112-91) from BD Pharmingen. The cytokine antibodies for flow cytometry were as follows: anti-IL-4 (11B11, BD Pharmingen), anti-IL-17a (TC11-18H10, BD Pharmingen), and anti-IFN-γ (XMG1.2, BD Pharmingen). Intracellular staining of Foxp3 or Bcl6 was performed using the Foxp3 Transcription Factor Staining Buffer Set (Invitrogen) according to the manufacturer’s protocols. For intracellular staining of cytokines, cells were stimulated for 4 h with PMA (Sigma-Aldrich, 5 ng/mL) plus ionomycin (Sigma-Aldrich, 0.5 μg/mL), and GolgiPlug Protein Trnsp Inhibitor (BD Pharmingen) for another 2 h. Cells were fixed with 4% formaldehyde after staining for cell surface markers (antibodies identified above) and permeabilized with 0.2% saponin (MP Biomedicals). Samples were acquired on a Fortessa or Celesta cytometer with FACSDiva (BD, Biosciences), and data were analyzed using FlowJo v.10 (BD, Biosciences).

### Cell purification and differentiation in vitro

Naive CD4^+^ T cells were enriched using a CD4^+^ T-cell enrichment isolation kit (STEMCELL Technologies) according to the manufacturer’s instructions and then sorted by 7-AAD^–^CD4^+^CD44^lo^CD62L^hi^CD25^–^CD4^+^ T cells on the AriaIII or AriaFusion system (BD, Biosciences). The sorted naive CD4^+^ T cell population was routinely more than 98% pure. Purified naive CD4^+^ T cells were stimulated for 2 d with anti-CD3 (precoated, 5 μg/mL, 145-2C11, BD Pharmingen) and anti-CD28 (in medium, 2 μg/mL, 37.51, BD Pharmingen) in complete T medium (RPMI 1640 with 10% heat-inactivated FCS, 2 mM L-glutamine, 1% penicillin-streptomycin, and 50 μM 2-mercaptoethanol). Then, these cells were expanded for another 2 d in T cell medium in the presence of 100 U/mL hIL-2. For different CD4^+^ T cell subset differentiation, naive CD4^+^ T cells were stimulated with anti-CD3 and anti-CD28 (identified above) in the presence of different combinations of cytokines as follows: for T_H_0 cell differentiation, hIL-2 (50 U/mL, Peprotech), anti-IFN-γ (10 μg/mL, Invitrogen), and anti-IL-4 (10 μg/mL, Invitrogen) were added; for T_H_1 cell differentiation, hIL-2 (50 U/mL, Peprotech), mIL-12 (10 ng/mL, Peprotech), and anti-IL-4 (10 μg/mL, Invitrogen) were added; for T_H_2 differentiation, hIL-2 (50 U/mL, Peprotech), mIL-4 (10 ng/mL, Peprotech), and anti-IFN-γ (10 μg/mL, Invitrogen) were added; for T_H_17 differentiation, mIL-6 (20 ng/mL, Peprotech), mIL-23 (10 ng/mL, R&D Systems), mIL-1β (10 ng/mL, R&D system), hTGF-β (1 ng/mL, R&D system), anti-IL-4 (10 μg/mL, Invitrogen), and anti-IFN-γ (10 μg/mL, Invitrogen) were added; for iT_reg_ differentiation, hIL-2 (50 U/mL, Peprotech), hTGF-β (2 ng/mL, R&D Systems), anti-IL-4 (10 μg/mL, Invitrogen), and anti-IFN-γ (10 μg/mL, Invitrogen) were added; for T_FH-like_ differentiation, mIL-6 (10 ng/mL, Peprotech), mIL-21 (10 ng/mL, R&D Systems), anti-IL-4 (10 μg/mL, Invitrogen), anti-IFN-γ (10 μg/mL, Invitrogen), and anti-TGF-β (10 μg/mL, R&D system) were added. Following surface staining, intracellular staining was performed (identified above), with IFN-γ^+^ cells as T_H_1 cells, IL-4^+^ cells as T_H_2 cells, IL-17a^+^ cells as T_H_17 cells, and Foxp3^+^ cells as iT_regs_.

### Retroviral transduction

The ORFs of *Bcl6* was cloned into the retroviral vector MSCV-IRES-GFP. The *Bcl6*^*5KR*^ mutation plasmids were constructed using the ClonExpress Ultra One Step Cloning Kit (Vazyme, C115-01). Retroviral plasmids containing sequences encoding *Bcl6* were produced in Plat-E cells (cultured in DMEM with 10% heat-inactivated FCS and 1% penicillin-streptomycin). Plat-E supernatant containing retroviruses was collected 48 h after transfection. For transduction of retrovirus, purified naive CD4^+^ T cells were activated for approximately 28 h with anti-CD3 (5 μg/mL) and anti-CD28 (5 μg/mL) in plates pre-coated with a hamster IgG antibody (20 μg/mL) in complete T medium (described above). Cells were transduced with retrovirus-containing supernatant with polybrene (8 μg/mL) plus hIL2 (100 U/mL) and centrifuged for 1.5 h at 1800 rpm. After 20 h of culture, the transduced CD4^+^ T cells were washed from the plates and cultured with a complete T-cell medium with hIL2 (100 U/mL) for another 2 d before sorting.

### Adoptive transfer

Naive CD4^+^ T cells obtained from OT-II *Cd4-cre*^*+/-*^ *Bcl6*^*fl/fl*^ mice were stimulated with anti-CD3 and anti-CD28 (identified above) for 28 h, and transduced with retroviruses expressing GFP alone (RV), WT Bcl6 (Bcl6^WT^), or 5KR mutant Bcl6 (Bcl6^5KR^). GFP^+^ T cells were sorted and washed twice with ice-cold 1× PBS. A total of 1 × 10^6^ purified cells were transferred into 6-week-old C57BL/6 recipient mice. After 1 d of rest, these recipient mice were immunized by intraperitoneal injection of 100 μg NP_14_-OVA (LGC Biosearch Technologies) in alum (Thermo Fisher Scientific).

### Immunoprecipitation and immunoblot analysis

The ORFs of *Trim37* was cloned into the pcDNA3.0 plasmids. The Trim37 mutation and truncation plasmids were constructed using the ClonExpress Ultra One Step Cloning Kit. Immunoprecipitation and immunoblot analysis were performed using standard protocols. Briefly, HEK293T cells were transfected (HighGene Transfection Reagent, ABclonal) with various combinations of pcDNA3.0 plasmids. At 24 h after transfection, the cells were washed with cold PBS twice, and lysates of the cells were prepared in RIPA lysis buffer (50 mM Tris base, 150 mM NaCl, 1% NP-40, 1 mM EDTA, 0.25% sodium deoxycholate, 0.1% SDS) containing 1× Protease Inhibitor Cocktail (Roche). For the in vitro ubiquitination assay, 20 mM MG132 (Sigma-Aldrich) was added to the culture medium 2 h before harvesting the cells. Ten percent of the lysates were mixed with 2× SDS loading buffer referred to as input. Ninety percent of the lysates were incubated with the anti-Flag Protein A/G Plus-Agarose gel (M2, Sigma-Aldrich) overnight at 4 °C. The complexes were washed three times with RIPA lysis buffer and diluted with 2× SDS loading buffer. The immunoprecipitated samples were analyzed by immunoblotting. β-actin (I-19, Santa Cruz Biotechnology) was used as an internal control throughout. The antibodies against HRP-conjugated 6× His were obtained from Proteintech. The mouse anti-Bcl6 (K112-91) antibody was obtained from BD Biosciences. The rabbit anti-HA antibody and anti-Flag antibody were obtained from Sigma-Aldrich. The rabbit anti-Trim37 (13037-1-AP) antibody was obtained from Proteintech. The quantitative analysis of western blot data was performed using ImageJ software (NIH).

### Ubiquitination assay in the reconstituted *E. coli* system

The procedures were performed as previously described^40^. Briefly, the pACYC-Ub-HA-E1-E2, pACYC-Ub-HA-E1-E2-Trim37^WT^, and pACYC-Ub-HA-E1-E2-Trim37^C18R^ plasmids were cotransformed with the pET22b-Bcl6-His plasmid into competent *E. coli* BL21 cells. Monoclonal cells were picked after sequencing, cultured in LB medium, and induced with 0.25 mM IPTG at 16 °C for 16 h. The cells were pelleted by centrifugation and resuspended in 8 M urea lysis buffer (50 mM Tris-HCl, 50 mM Na_2_HPO_4_, 0.5% NP-40, 300 mM NaCl, 8 M urea, 20 mM imidazole, pH 8.0) for sonication for cell lysis. The supernatant was subjected to incubation with Ni-NTA affinity gel (Qiagen) for 4 h and washed 3 times with 8 M urea lysis buffer. The eluted proteins were analyzed by immunoblot analysis. For two-step enrichment of the proteins, Ni-NTA affinity gels were eluted with RIPA lysis buffer with 1 M imidazole before 9 volumes of RIPA lysis buffer were added. Then, the second eluted proteins were immunoprecipitated with anti-HA affinity gel (Sigma-Aldrich) and washed three times with RIPA lysis buffer. The eluted proteins were subjected to mass spectrum analysis.

### Confocal microscopy and histology

The procedures were performed as previously described^58^. Briefly, HEK293T cells were transfected with plasmids encoding HA-tagged Trim37 and Flag-tagged Bcl6 for 24 h. Then, these cells were fixed with 4% PFA in PBS and permeabilized with Triton X-100. After blocking with 10% FBS in PBS, these cells were stained with mouse anti-HA and rabbit anti-Flag antibodies, followed by Alexa Fluor 647 goat anti-rabbit IgG and Alexa Fluor 488 rat anti-mouse IgG. Nuclei were stained with DAPI. The fluorescent images were captured with a Leica TCS SP8 laser confocal microscope. On day 12 after influenza virus infection, lymph nodes and spleens were fixed with 4% PFA and 10% sucrose in PBS for 1 h at 4 °C. Then, fixed tissues were incubated overnight in 30% sucrose and embedded in OCT compound (Thermo Fisher Scientific). Cryosectioned tissues were blocked with 10% FBS and 1% Fc blocker (Invitrogen) and then stained with Alexa Fluor 647 anti-B220 (RA3-6B2, BD Biosciences) and Alexa Fluor 488 anti-GL7 (GL7, BioLegend) overnight at 4°C. Mounted sections were imaged on an Olympus FV3000 confocal microscope. Lungs were fixed with 4% PFA in PBS, embedded in paraffin, and stained with hematoxylin and eosin using standard protocols. Mounted sections were imaged on a Zeiss Scan.Z1 system.

### Bone marrow chimera experimentation

For generation of bone marrow chimeras, 1 × 10^7^ T-cell-depleted bone marrow cells were obtained from WT or *Trim37* mutant (*Trim37*^*C18R/C18R*^) mice and transferred into irradiated *Tcrb*^*-/-*^ (800 Rad) mice. For generation of mixed bone marrow chimeras, T-cell-depleted bone marrow cells were obtained from WT (CD45.1) or *Trim37* mutant (*Trim37*^*ko*^ CD45.2, *FIN*_*major*_ CD45.2, *Trim37*^*G322V/G322V*^ CD45.2) mice and mixed at a ratio of 1:1 before they were transferred into irradiated *Tcrb*^*-/-*^ (800 Rad) mice. Reconstituted bone marrow mice were challenged with influenza virus 10 weeks later.

### Enzyme-linked immunosorbent and viral plaque/microneutralization assay

The procedures used to measure HA-specific antibodies were previously described^53^. Briefly, 96-well plates (Nunc) were coated with 1 μg/mL HA protein (Influenza A H1N1 (A/Puerto Rico/8/1934) Haemagglutinin, SinoBiological) in Coating Buffer (0.1 M Na_2_CO_3_, 0.1 M NaHCO_3_, pH 9.5) at 4 °C overnight. HRP-conjugated goat anti-mouse IgG1 (Southern Biotechnology Associates), and HRP-conjugated anti-mouse IgG (R&D Systems) were used at 1:2000 to detect antigen-specific antibodies in serum. The procedures used in the microneutralization assay were previously described^53^. Briefly, MDCK cells were seeded into 96-well plates on day –1. Then, these cells were washed twice with PBS and incubated in DMEM with 2 μg/mL trypsin (T1426, Sigma-Aldrich) at Day 0. Serum samples were serially diluted 2-fold in 50 μL of DMEM and then mixed with 100 TCID50 of PR8 influenza virus in 50 μL of DMEM for 1 h at 37 °C. 1 h later, the virus-serum mixture was transferred to MDCK cells and incubated for 24 h. After 24 h of incubation, the supernatant was removed, the cells were washed twice with PBS and fixed in 80% acetone for 30 min, and viral antigen was detected by ELISAs with a polyclonal antibody against NP protein. The OD_450_ was recorded. Viral plaque assays were performed as previously described^57^.

### Quantitative real-time PCR analysis

The procedures used in this study were previously described^53^. Total RNA was prepared from cells using TRIzol reagent (Invitrogen). The purified RNA was quantified, and reverse transcribed by using HiScript III-RT SuperMix for the qPCR kit from Vazyme. The expression of mRNA was normalized to *Hprt* expression. qPCR primers are shown in Supplementary Table S3.

### Statistical analysis

No statistical methods were used to predetermine sample size. GraphPad Prism software was used for all statistical analysis except the genetic burden test. Statistical significance was determined by Fisher test, two-tailed paired or unpaired Student’s *t*-test and ANOVA as described in the figure legends. *P* values were considered significant when less than 0.05. ns, not significant, *P < 0.05, **P < 0.01, ***P < 0.001 and ****P < 0.0001. Data are mean ± SEM.

## Supplementary information

Supplementary information

## Data Availability

All data are available in the main text or the supplementary materials.

## Materials availability

Reagents generated in this study will be made by reasonable request to the lead contact with a complete materials transfer agreement.
