# Supplementary material for: The TRIM37 variants in Mulibrey nanism patients paralyze follicular helper T cell differentiation
Source: Cell Discov. 2023 Aug 1;9:82. doi: 10.1038/s41421-023-00561-z (PMC10394018; doi:10.1038/s41421-023-00561-z)
Supplement: Supplementary file 1 — Supplementary information [file 41421_2023_561_MOESM1_ESM.pdf]

Supplementary information for

The *Trim37* variants in Mulibrey nanism patients paralyze follicular helper T cell differentiation

Wangpeng Gu[1,2,12], Jia Zhang[2,12], Qing Li[2,12], Yaguang Zhang[2,12,*], Xuan Lin[3,7,12], Bingbing Wu[5,12], Qi Yin[2,12], Jinqiao Sun[8], Yulan Lu[5], Xiaoyu Sun[2], Caiwei Jia[2], Chuanyin Li[2], Yu Zhang[3], Meng Wang[3], Xidi Yin[2], Su Wang[1,2], Jiefang Xu[1,2], Ran Wang[1,2], Songling Zhu[1,2], Shipeng Cheng[2], Shuangfeng Chen[2], Lian Liu[2], Lin Zhu[2], Chenghua Yan[2], Chunyan Yi[2], Xuezhen Li[2], Qiaoshi Lian[2], Guomei Lin[2], Zhiyang Ling[2], Liyan Ma[2], Min Zhou[9], Kuanlin Xiao[9], Ze Chen[10], Dangsheng Li[2], Haiming Wei[1], Ronggui Hu[2,*], Wenhao Zhou[5,6,*], Lilin Ye[4,11,*], Haikun Wang[3,*], Jinsong Li[2,*] and Bing Sun[2,*]

Supplementary Figures

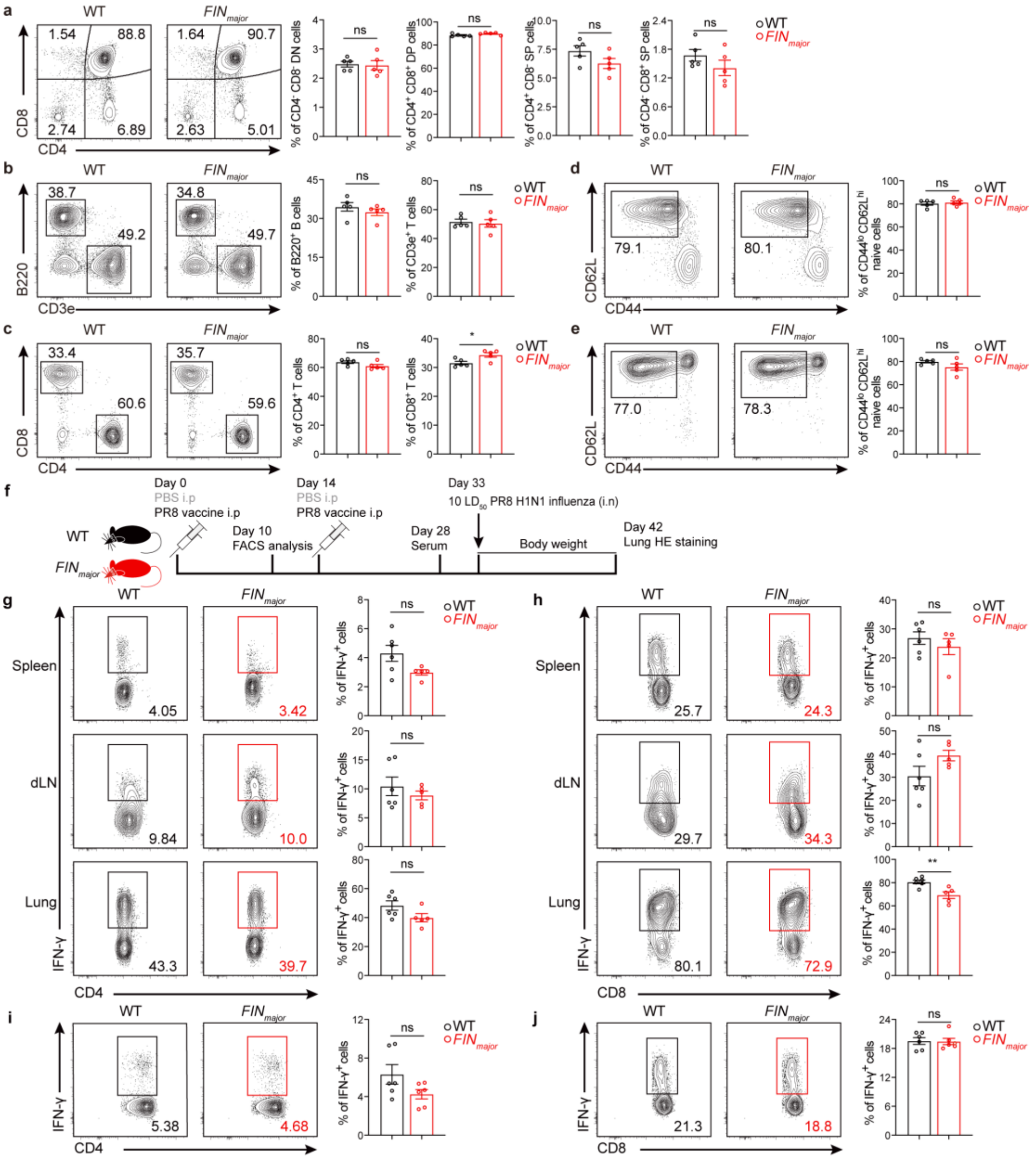

Fig. S1 ***Trim37*** **deficiency does not impair adaptive immune system development and cellular immune responses. a** Representative flow cytometry plots illustrate the frequency of CD4$^+$ SP, CD8$^+$ SP, CD4$^+$ CD8$^+$ DP, and CD4$^-$ CD8$^-$ DN cells as a percentage of live cells in the thymus. **b** Representative flow cytometry plots illustrate the frequency of CD3e$^+$ T cells and B220$^+$ B cells as a percentage of live cells in the spleen. **c** Representative flow cytometry plots illustrate the frequency of CD4$^+$ T cells and CD8$^+$ T cells as a percentage of CD3e$^+$ T cells in the spleen. **d** Representative flow cytometry plots illustrate the frequency of CD44^{lo} CD62L^{hi} naive T cells as a percentage of CD4$^+$ T cells in the spleen. **e** Representative flow cytometry plots

illustrate the frequency of $CD44^{lo}$ $CD62L^{hi}$ naive T cells as a percentage of $CD8^+$ T cells in the spleen. **f** Schematic experimental design of the vaccine-challenge model. **g–h** WT (n = 6) and FIN_{major} (n = 5) mice were infected intranasally with PR8 H1N1 influenza virus (0.5 LD_{50}). These mice were sacrificed on Day 12 after infection, and spleen, lung draining lymph node (dLN), and lung tissue were collected for single-cell suspensions. The cells were treated with PMA plus ionomycin for 4 hours and then incubated with BFA for another 2 hours, and intracellular cytokine staining was performed. **g** Representative flow cytometry plots illustrate the frequency of IFN-γ^+ T_H1 cells as a percentage of $CD4^+$ T cells in the spleen (**up**), dLN (**middle**), and lung (**down**). **h** Representative flow cytometry plots illustrate the frequency of IFN-γ^+ cytotoxic $CD8^+$ T cells as a percentage of $CD8^+$ T cells in the spleen (**up**), dLN (**middle**), and lung (**down**). **i–j** WT (n = 5) and FIN_{major} (n = 5) mice were immunized with PR8 vaccine for 10 days, representative flow cytometry plots illustrate the frequency of IFN-γ^+ T_H1 cells (**i**) as a percentage of $CD4^+$ T cells in the spleen and the frequency of IFN-γ^+ cytotoxic $CD8^+$ T cells as a percentage of $CD8^+$ T cells in the spleen (**j**). Data are representative of at least three independent experiments, and were analysed by two-tailed unpaired Student's *t*-test. Data are mean ± s.e.m. * $P< 0.05$, ** $P < 0.01$, *** $P < 0.001$ and ns, not significant.

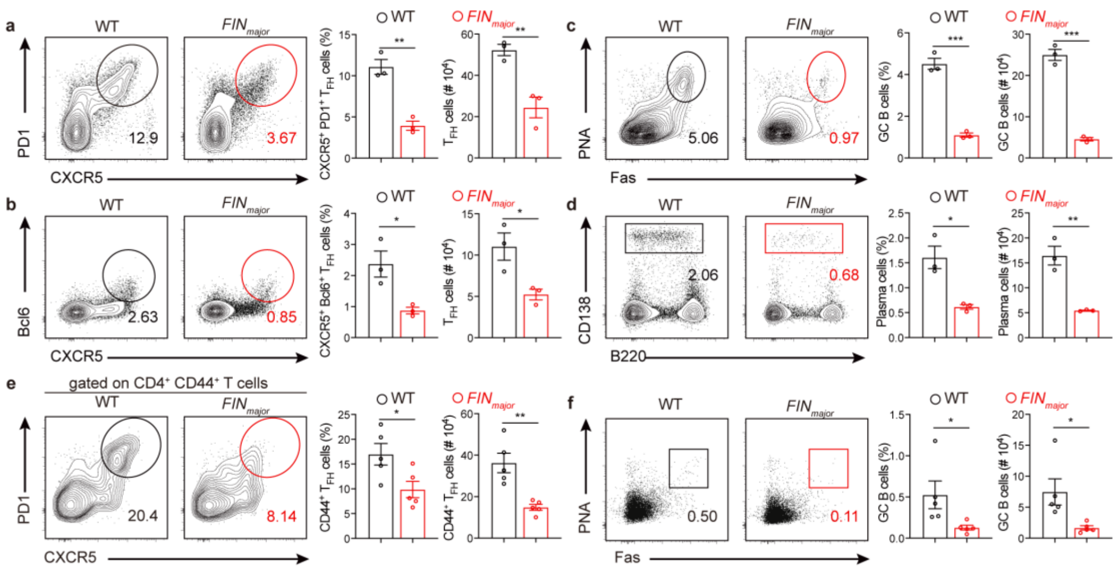

Fig. S2 Trim37 is required for T_{FH} cell differentiation. a–d WT (n=3) and *FIN*$_{major}$ (*n* = 3) mice were infected intranasally with PR8 H1N1 influenza virus (0.5 LD_{50}). These mice were sacrificed on Day 12 after infection. **a** Representative flow cytometry plots illustrate the frequency of $CXCR5^+$ $PD1^+$ T_{FH} cells as a percentage of $CD4^+$ $Foxp3^-$ T cells in the spleen, quantification of $CXCR5^+$ $PD1^+$ T_{FH} cells. **b** Representative flow cytometry plots illustrate the frequency of $CXCR5^+$ $Bcl6^+$ T_{FH} cells as a percentage of $CD4^+$ $Foxp3^-$ T cells in the spleen, quantification of $CXCR5^+$ $Bcl6^+$ T_{FH} cells. **c** Representative flow cytometry plots illustrate the frequency of Fas^+ PNA^+ GC B cells as a percentage of $B220^+$ B cells in the spleen, quantification of GC B cells. **d** Representative flow cytometry plots illustrate the frequency of $B220^{lo}$ $CD138^{hi}$ plasma cells as a percentage of live cells in the spleen, quantification of plasma cells. **e, f** Flow cytometry analysis of the splenocytes obtained from uninfected WT (*n* = 5) and *FIN*$_{major}$ (*n* = 5) mice. **e** Representative flow cytometry plots illustrate the frequency of $CXCR5^+$ $PD1^+$ T_{FH} cells as a percentage of activated $CD4^+$ T cells in the spleen, quantification of $CXCR5^+$ $PD1^+$ T_{FH} cells. **f** Representative flow cytometry plots illustrate the frequency of Fas^+ PNA^+ GC B cells as a percentage of $B220^+$ B cells in the spleen, quantification of GC B cells. Data are representative of at least three independent experiments, and were analysed by two-tailed unpaired Student's *t*-test. Data are mean ± s.e.m. *$P < 0.05$, **$P < 0.01$, ***$P < 0.001$ and ns, not significant.

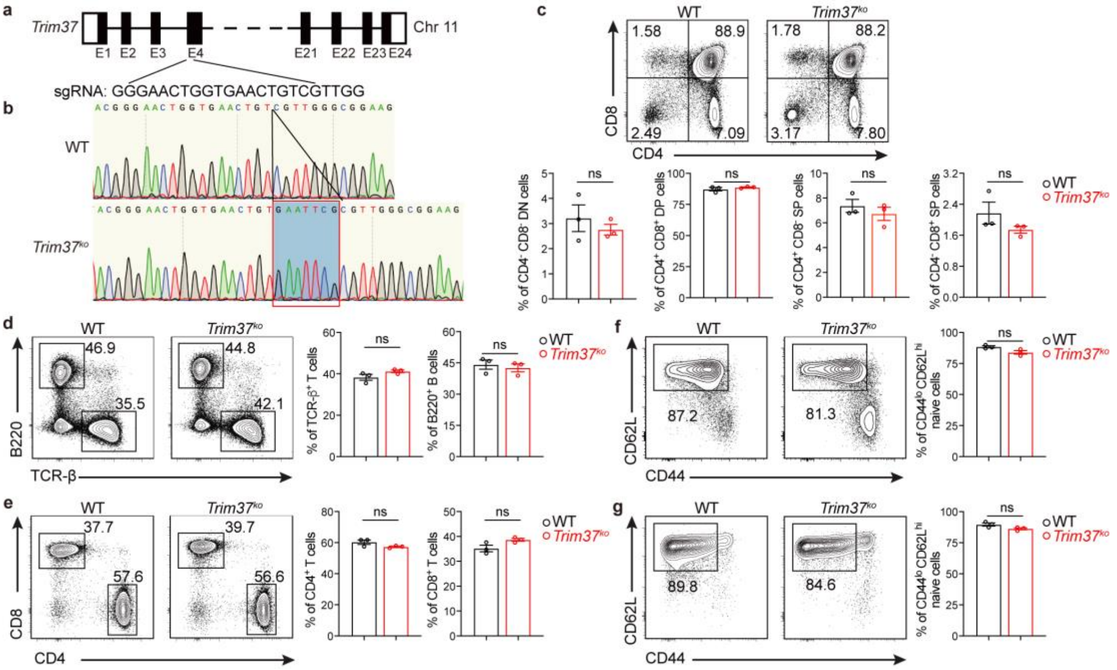

Fig. S3 Normal adaptive immune system development in *Trim37*^{ko} mice. a The schematic design of *Trim37*^{ko} mice. We inserted a 7 bp oligonucleotide (GAATTCG) into the *Trim37* gene locus (exon 4), which led to Trim37 truncation. **b** Sanger sequence analysis of the WT and *Trim37*^{ko} mice. **c**–**g** Flow cytometric analysis of adaptive immune system development in WT and *Trim37*^{ko} mice (WT, *n* = 3, *Trim37*^{ko}, *n* = 3). **c** Representative flow cytometry plots illustrate the frequency of $CD4^+$ SP, $CD8^+$ SP, $CD4^+$ $CD8^+$ DP, and $CD4^-$ $CD8^-$ DN cells as a percentage of live cells in the thymus. **d** Representative flow cytometry plots illustrate the frequency of TCR-β^+ T cells and $B220^+$ B cells as a percentage of live cells in the spleen. **e** Representative flow cytometry plots illustrate the frequency of $CD4^+$ T cells and $CD8^+$ T cells as a percentage of TCR-β^+ T cells in the spleen. **f** Representative flow cytometry plots illustrate the frequency of $CD44^{lo}$ $CD62L^{hi}$ naive T cells as a percentage of $CD4^+$ T cells in the spleen. **g** Representative flow cytometry plots illustrate the frequency of $CD44^{lo}$ $CD62L^{hi}$ naive T cells as a percentage of $CD8^+$ T cells in the spleen. Data are representative of at least three independent experiments, and were analysed by two-tailed unpaired Student's *t*-test. Data are mean ± s.e.m. **P* < 0.05, ***P* < 0.01, ****P* < 0.001 and ns, not significant.

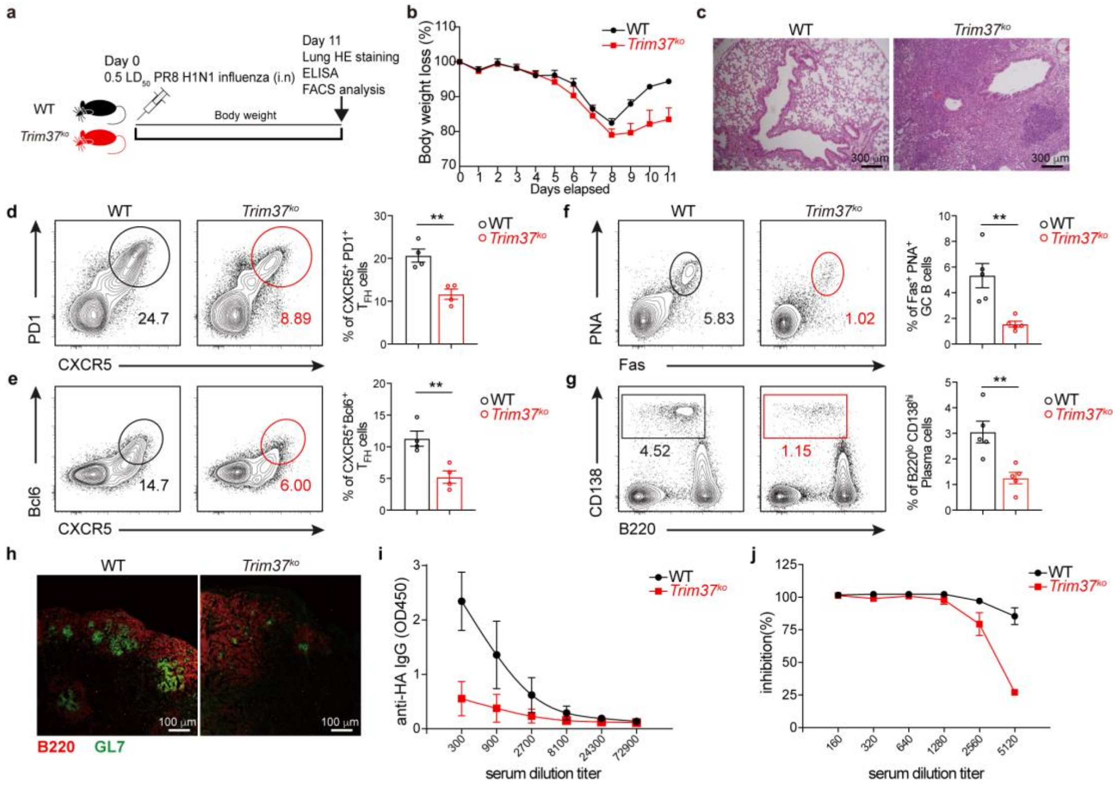

Fig. S4 *Trim37*^{ko} mice are susceptible to influenza virus infection. a WT (n = 4) and *Trim37*^{ko} (n = 4) mice were infected intranasally with PR8 H1N1 influenza virus (0.5 LD$_{50}$). These mice were sacrificed on Day 11 after infection. **b** The body weight of the WT and *Trim37*^{ko} mice was monitored daily after influenza virus infection. **c** Histopathological analysis of lung tissue was performed by HE staining. **d–h** Representative flow cytometry plots illustrate the frequency of CXCR5$^+$ PD1$^+$ (**d**) and CXCR5$^+$ Bcl6$^+$ T$_{FH}$ cells (**e**) as a percentage of CD4$^+$ T cells in the lung draining lymph nodes. **f** Representative flow cytometry plots illustrate the frequency of Fas$^+$ PNA$^+$ GC B cells as a percentage of B220$^+$ B cells in the lung draining lymph nodes. **g** Representative flow cytometry plots illustrate the frequency of B220^{lo} CD138^{hi} plasma cells as a percentage of live cells in the lung draining lymph nodes. **h** Confocal microscopy of the lymph node's germinal centre (B220$^+$ GL7$^+$). **i** Viral-specific anti-HA IgG in the sera obtained from infected WT and *Trim37*^{ko} mice was measured by ELISAs. **j** In vitro microneutralization against PR8 influenza virus in the sera obtained from infected WT and *Trim37*^{ko} mice. Data are representative of at least three independent experiments, and were analysed by two-tailed unpaired Student's *t*-test. Data are mean ± s.e.m. **P* < 0.05, ***P* < 0.01, ****P* < 0.001 and ns, not significant.

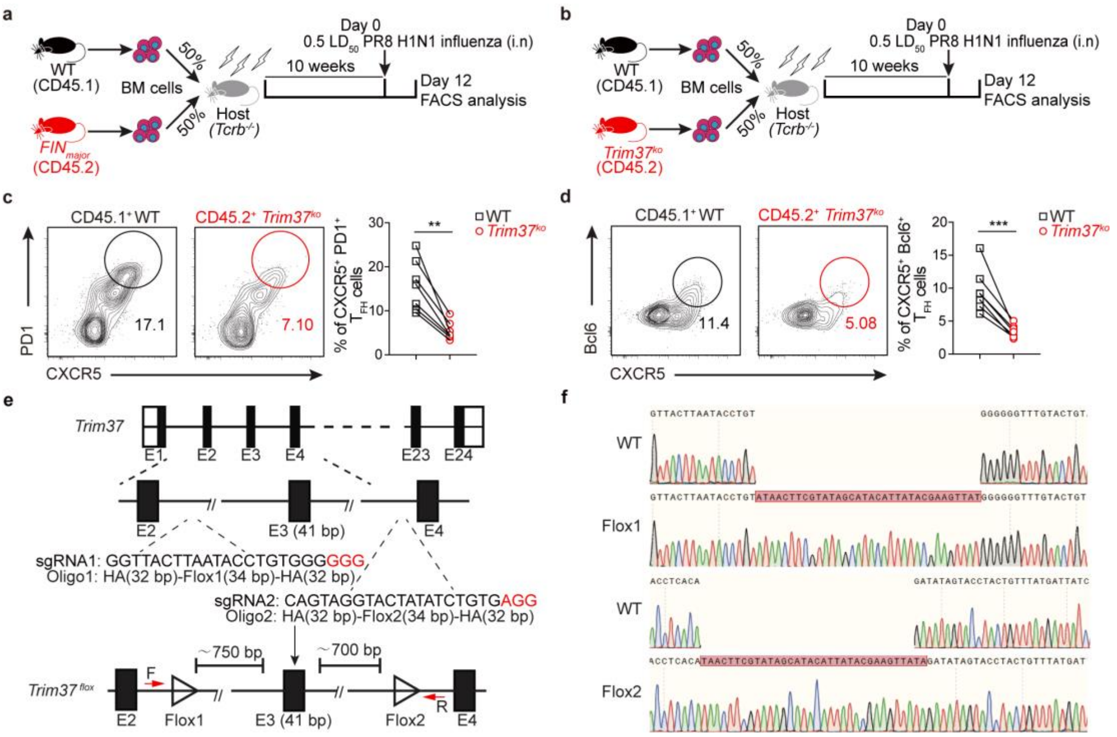

Fig. S5 Trim37 promotes T_{FH} differentiation in a T-cell-intrinsic manner. a Schematic illustration of the WT and *FIN_{major}* mixed bone marrow chimaera model. These bone marrow chimeric mice were infected intranasally with PR8 H1N1 influenza virus (0.5 LD_{50}) and sacrificed on day 12 after infection. **b** Schematic illustration of the WT and *$Trim37^{ko}$* mixed bone marrow chimaera model. These bone marrow chimeric mice were infected intranasally with PR8 H1N1 influenza virus (0.5 LD_{50}) and sacrificed on day 12 after infection. **c, d** Representative flow cytometry plots showing the frequency of $CD45.1^+$ (WT, n = 7) or $CD45.2^+$ (*$Trim37^{ko}$*, n = 7) $CXCR5^+$ $PD1^+$ T_{FH} cells (**c**) and $CXCR5^+$ $Bcl6^+$ T_{FH} cells (**d**) as a percentage of $CD4^+$ $Foxp3^-$ T cells in the spleen. **e** The schematic design of *$Trim37^{flox}$* mice. **f** Sanger sequencing of WT and *$Trim37^{flox}$* mice. Data are representative of at least three independent experiments, and were analysed by two-tailed paired Student's *t*-test. Data are mean ± s.e.m. *P < 0.05, **P < 0.01, ***P < 0.001 and ns, not significant.

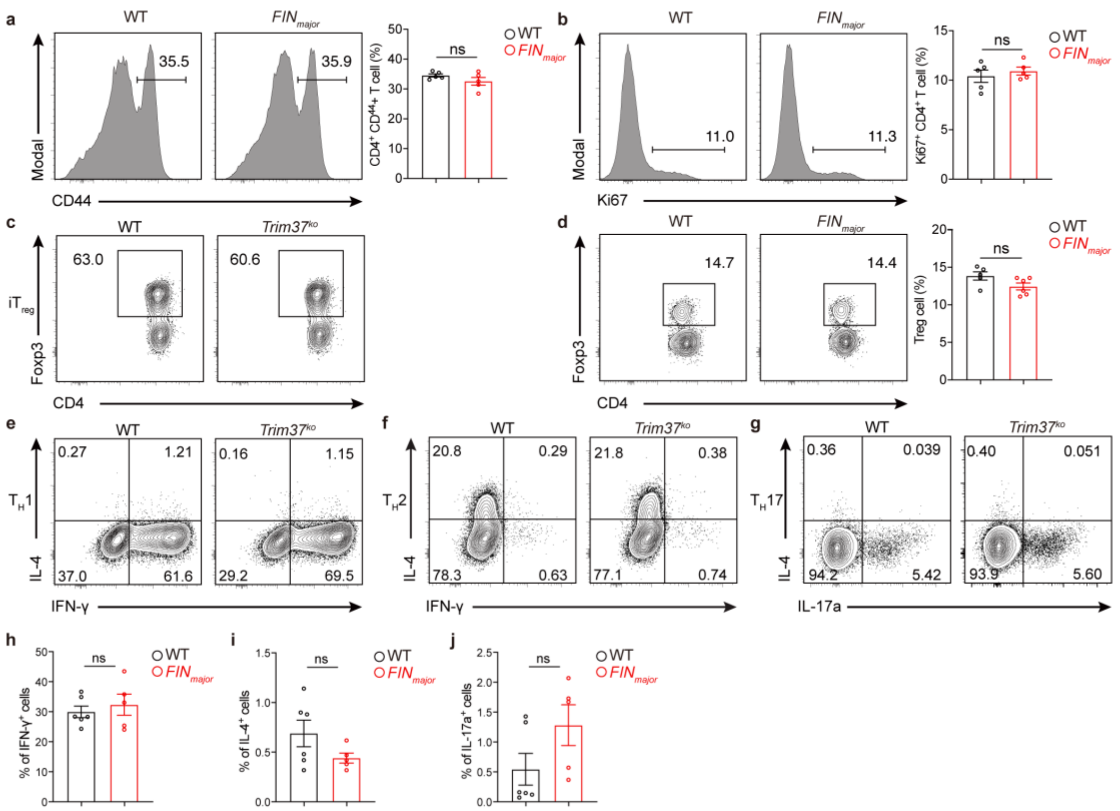

Fig. S6 T-cell activation, proliferation and *in vitro* differentiation. a, b Representative flow cytometry plots illustrate the frequency of CD44$^+$ (**a**) and Ki67$^+$ cells (**b**) as a percentage of CD4$^+$ T cells in the lung draining lymph nodes of WT and *FIN$_{major}$* mice at day 12 after influenza virus infection. **c** Flow cytometry analysis of CD4$^+$ T cells from WT and *Trim37^{ko}* mice at day 4 after culturing under iT$_{reg}$ condition. **d** Representative flow cytometry plots illustrate the frequency of Foxp3$^+$ cells as a percentage of CD4$^+$ T cells in the lung draining lymph nodes at day 12 after influenza virus infection. **e–g** Flow cytometry analysis of CD4$^+$ T cells from WT and *Trim37^{ko}* mice at day 4 after culturing under T$_H$1, T$_H$17, and iT$_{reg}$ conditions. **h–j** The frequency of IFN-γ$^+$ (**h**), IL-4$^+$ (**i**), and IL-17a$^+$ cells (**j**) as a percentage of CD4$^+$ CD44$^+$ T cells in the lung draining lymph nodes of WT and *FIN$_{major}$* mice at day 12 after influenza virus infection. Data are representative of at least three independent experiments.

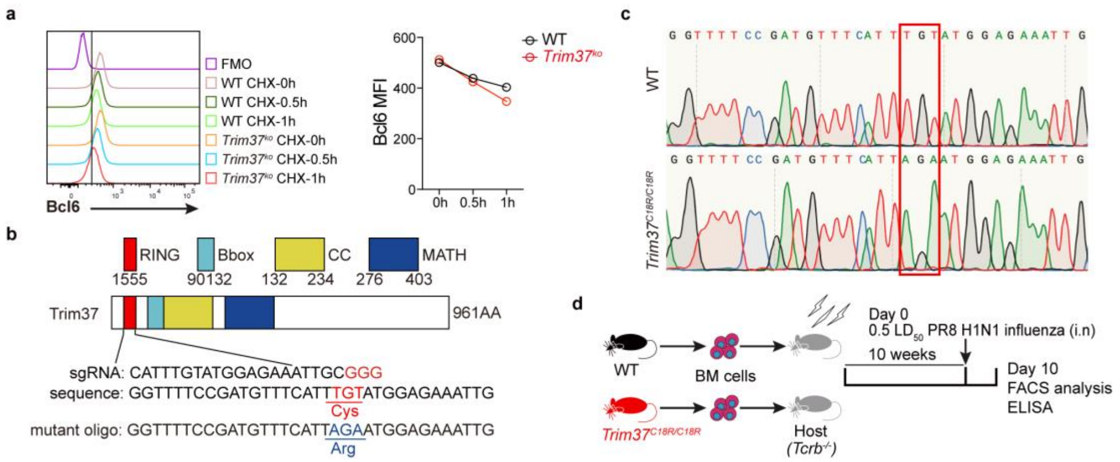

Fig. S7 Construction of *Trim37*$^{C18R/C18R}$ mice. a Flow cytometry analysis of Bcl6 expression in CHX-treated WT and *Trim37*^{ko} $T_{FH\text{-like}}$ cells (left). Quantification of Bcl6 MFI (right). (FMO, **F**luorescence **M**inus **O**ne control). **b** The schematic design of *Trim37*$^{C18R/C18R}$ mice. **c** Sanger sequencing of WT and *Trim37*$^{C18R/C18R}$ mice. **d** Schematic illustration of the WT and *Trim37*$^{C18R/C18R}$ bone marrow chimera model. These bone marrow chimeric mice were infected with influenza virus for 10 days, and the spleen and serum were collected for the following assays. Data are representative of at least three independent experiments.

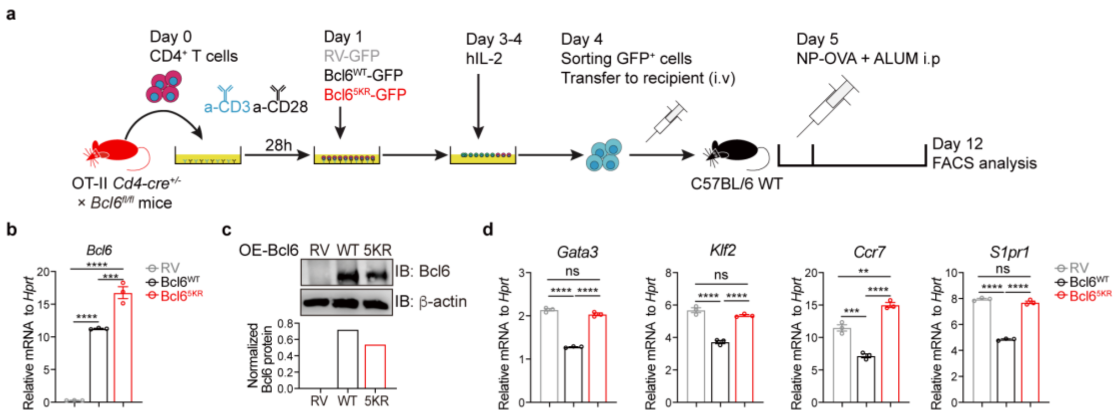

Fig. S8 Trim37 ubiquitinates Bcl6 at the K227, K302, K327, K535 and K689 residues. a The schematic pipeline of the Bcl6 rescue experiment. **b** The mRNA expression of *Bcl6* was detected in T_H0 cells transduced with RV, Bcl6^{WT} or Bcl6^{5KR}-expressing retroviruses. **c** Immunoblot analysis of Bcl6 protein in T_H0 cells transduced with RV, Bcl6^{WT} or Bcl6^{5KR}-expressing retroviruses. Relative Bcl6 protein, normalized to actin (down). **d** The mRNA expression of *Gata3*, *Klf2*, *Ccr7* and *S1pr1* was detected in T_H0 cells transduced with RV, Bcl6^{WT} or Bcl6^{5KR}-expressing retroviruses. Data are representative of at least three independent experiments, and were analysed by One-way ANOVA (**b, d**). Data are mean ± s.e.m. *$P < 0.05$, **$P < 0.01$, ***$P < 0.001$, ****$P < 0.0001$ and ns, not significant.

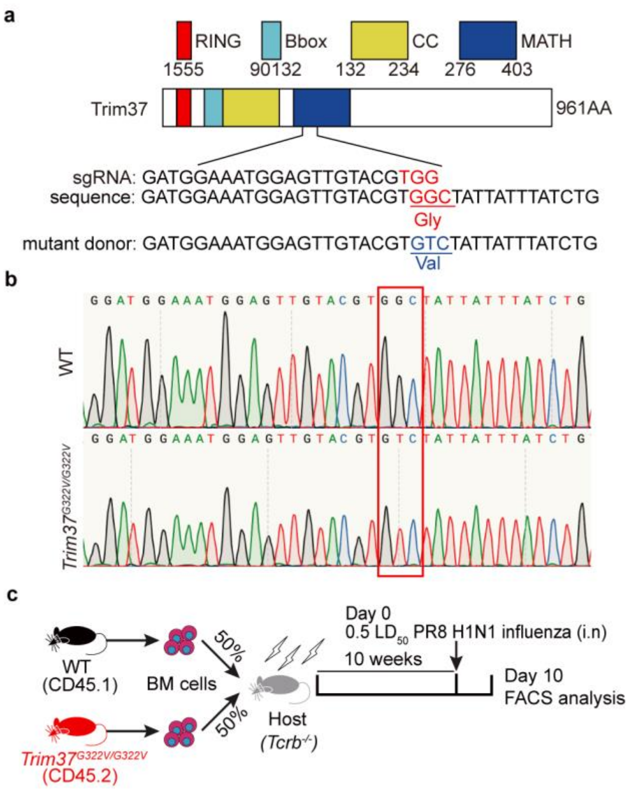

Fig. S9 Construction of *Trim37*$^{G322V/G322V}$ mice. a The schematic design of *Trim37*$^{G322V/G322V}$ mice. **b** Sanger sequencing of WT and *Trim37*$^{G322V/G322V}$ mice. **c** Scematic illustration of the WT and *Trim37*$^{G322V/G322V}$ mixed bone marrow chimaera model. These bone marrow chimeric mice were infected with influenza virus for 10 days, and the spleen and serum were collected for the following assays. Data are representative of at least three independent experiments.

Supplementary Tables

Supplementary Table 1 Types of VEPANNOVAR annotated variants and classification

ANNOVAR annotated variant mutation type	VEP annotated variant mutation type	Impact	Score	Mutation Type Group
splicing	splice acceptor variant	High	2	PTV
	splice donor variant	High	2	PTV
stopgain	stop gained	High	2	PTV
frameshift insertion	frameshift variant	High	2	PTV
frameshift deletion		High	2	PTV
frameshift block substitution		High	2	PTV
stoploss	start lost	High	2	PTV
	stop lost	High	2	PTV
nonsynonymous SNV	missense variant	Moderate	1	MIS
nonframeshift deletion	inframe deletion	Moderate	1	MIS
nonframeshift insertion	inframe insertion	Moderate	1	MIS
synonymous SNV	synonymous variant	Low	0	SYN
	splice region variant	Low	0	NON
intronic	start retained variant	Low	0	NON
	stop retained variant	Low	0	NON
	intron variant	Modifier	0	NON
ncRNA_exonic	non coding transcript exon variant	Modifier	0	NON
UTR3	3 prime UTR variant	Modifier	0	NON
UTR5	5 prime UTR variant	Modifier	0	NON
upstream	upstream_gene_variant	Modifier	0	NON
downstream	downstream_gene_variant	Modifier	0	NON

Supplementary Table 2 Detailed information of the burden test for recurrent infection

Gene symbol	Count in Case	Count in BG	#Case	#BG	*P* value	OR	Rank
Nt5e	18	303	447	15883	0.00334	2.16	1
Stk4	13	195	447	15883	0.00489	2.41	2
Tbxas1	15	259	447	15883	0.0087	2.09	3
Trim37	14	249	447	15883	0.0138	2.03	4
Sos1	12	204	447	15883	0.0161	2.12	5
Hpse2	11	180	447	15883	0.0163	2.2	6
Afp	13	232	447	15883	0.0177	2.02	7
Scn4a	11	187	447	15883	0.0207	2.12	8
Rara	10	163	447	15883	0.0209	2.21	9
Cyp2d6	10	175	447	15883	0.0313	2.05	10
Fkbp10	10	179	447	15883	0.0355	2.01	11

Supplementary Table 3 List of primers and oligos in this study

sgRNA sequence	Sequence (5'-3')
FINmajor-sgRNA	TTCCTAGAAGATAAACACGAAGG
Trim37-KO-sgRNA	GGGAACTGGTGAACTGTCGTTGG
Trim37-C18R-sgRNA	TTTCCGATGTTTCATTTGTATGG
Trim37-3xflag-sgRNA	CTCTGCTCATCCATTGCCTCCGG
Trim37-flox-sgRNA1	GGTTACTTAATACCTGTGGGGGG
Trim37-flox-sgRNA2	CAGTAGGTACTATATCTGTGAGG
Trim37-G322V-sgRNA	GATGGAAATGGAGTTGTACGTGG

Oligo donors homology directed repair (HDR)	Sequence (5'-3')
FINmajor-donor	ACAATTTGTCCTTCGTGTTTATCTT<br>CTGGGAAAGAAATGTAGAAGCTGT<br>AAGAAATGCAAAGGACGAGCGTG<br>TTCGGGAAATTAGGAATG
Trim37-C18R-donor	CTCTTTTGTTTTCAGAGCATTGCT<br>GAGGTTTTCCGATGTTTCATTAG<br>AATGGAGAAATTGCGGGATGCT<br>CGACTGTGTCCTCATTGCTCCAA<br>GCTCTGTT
Trim37-flox1-donor	ATCACAGGTCTATTGCAGTATTT<br>TTCTGGAAGGTTACTTAATACCT<br>GTATAACTTCGTATAGCATACAT<br>TATACGAAGTTATGGGGGGTTTG<br>TACTGTACTTCTTATTAGTCCGTT<br>CCTCAGTAAATGAC
Trim37-flox2-donor	AAGTGGTCATGTATATTCTTACA<br>TACGTTTTATTTTTTTAAACCTCA<br>CATAACTTCGTATAGCATACATTA<br>TACGAAGTTATAGATATAGTACCT<br>ACTGTTTATGATTATCTTGCTTGTT<br>GTTTATTTAC

Primers for genotyping	Sequence (5’-3’)
FINmajor-check-F	TCTTTCACCTTACTGACCCAAA
FINmajor-check-R	TGGCAACAGAATGCAAGCAC
Trim37-KO-check-F	AGCTTCAGTTCTGTAACCTTAGTG
Trim37-KO-check-R	GCATGTGGCCCCAGGATAAA
Trim37-C18R-check-F	TCAGTGTAATCTGGGACCCT
Trim37-C18R-check-R	AAAAAGGGGAGGGCATGGTT
Trim37-3xflag-check-F	TGGCGAAATGCGGGGTAG
Trim37-3xflag-check-R	ATAGAAAAGGAGCCCAGCCG
Trim37-flox1-check-F	TTTTCCGTTTGCGCCTTCC
Trim37-flox1-check-R	GTCATTTACTGAGGAACGGACT
Trim37-flox2-check-F	TGGATAGGCATGCTATTCTTGTATG
Trim37-flox2-check-R	AGCGAGGTTAACCGGAAGGA
Bcl6-TST-Check-F	CAGCTAACACTTGGCTTCTGC
Bcl6-TST-Check-R	TGCTGACAGGGTTTGCCTTAC
Trim37-G322V-check-F	CTGTTTGTTGGCTTCATTGT
Trim37-G322V-check-R	AAGAGTTTCGACACAGCAAT
Cd4-Cre-check-F	CGGTCGATGCAACGAGTGATGAGG
Cd4-Cre-check-R	CCAGAGACGGAAATCCATCGCTCG
OT-II-check-F	GCTGCTGCACAGACCTACT
OT-II-check-R	CAGCTCACCTAACACGAGGA
Bcl6-flox-F	GTGTCCTGGGGTTACAGGTG
Bcl6-flox-R	CCTGTCCTGCCTACCCATAG

Primers for qPCR	Sequence (5'-3')
Hprt-F	TGCTCGAGATGTCATGAAGGA
Hprt-R	CAGAGGGCCACAATGTGATG
Cxcr5-F	GACCTTCAACCGTGCCTTTCTC
Cxcr5-R	GAACTTGCCCTCAGTCTGTAATCC
Trim37-F	TCCAAGCTCTGTTGTTTCAGC
Trim37-R	TTCCGCCCAACGACAGTTC
Bcl6-F	CCGGCACGCTAGTGATGTT
Bcl6-R	TGTCTTATGGGCTCTAAACTGCT
Pdcd1-F	GCTCACTTCAGGTTTACCACAAGC
Pdcd1-R	GCCCAACAGTAGGATTCAGGAGAC
Prdm1-F	TGGAGGATCTGACCCGAATC
Prdm1-R	CGCTGATGTCGAACCTCTCAA
Il7r-F	GCGGACGATCACTCCTTCTG
Il7r-R	AGCCCCACATATTTGAAATTCCA
Ifngr1-F	CTGGCAGGATGATTCTGCTGG
Ifngr1-R	GCATACGACAGGGTTCAAGTTAT
Runx3-F	CAGGTTCAACGACCTTCGATT
Runx3-R	GTGGTAGGTAGCCACTTGGG
Gata3-F	CATTAGCGTTCCTCCTCCAG
Gata3-R	CTTATCAAGCCCAAGCGAAG
Klf2-F	GCCTTATCATTGCAACTGGGA
Klf2-R	TCAGAGCGCGCGAACTTC
Ccr7-F	TGTACGAGTCGGTGTGCTTC

**Primers for qPCR**	**Sequence (5’-3’)**
Ccr7-R	GGTAGGTATCCGTCATGGTCTTG
S1pr1-F	GAGCACGGTGGAGCAGCTAG
S1pr1-R	ATCATGGGCTGGAACTGCAT
